# Psychometric assessment of EQ-5D-5L and ReQoL measures in patients with anxiety and depression: construct validity and responsiveness

**DOI:** 10.1007/s11136-021-02833-1

**Published:** 2021-04-09

**Authors:** Matthew Franklin, Angel Enrique, Jorge Palacios, Derek Richards

**Affiliations:** 1 Health Economics and Decision Science (HEDS), School of Health and Related Research (ScHARR), University of Sheffield, West Court, 1 Mappin Street, Sheffield, S1 4DT UK; 2 Cinical Research & Innovation, SilverCloud Health, Dublin, Ireland UK; 3 E-mental Health Research Group, School of Psychology, University of Dublin, Trinity College, Dublin, Ireland UK

**Keywords:** EQ-5D-5L, ReQoL-10, ReQoL-UI, Anxiety, Depression, Psychometrics, Economic evaluation

## Abstract

**Purpose:**

Generic health measures have been questioned for quantifying mental-health-related outcomes. In patients with anxiety and/or depression, our aim is to assess the psychometric properties of the preference-based EQ-5D-5L (generic health) and ReQoL-UI (recovery-focussed quality of life) for economic evaluation against the PHQ-9 (depression) and GAD-7 (anxiety). EQ-5D-5L anxiety/depression item and ReQoL-10 are also assessed.

**Methods:**

A 2:1 (intervention: control) randomised controlled trial collected measures at baseline and 8 weeks post baseline; in the intervention arm, data were also collected 3, 6, 9, and 12-months post baseline. EQ-5D-5L preference-based scores were obtained from the value set for England (VSE) and ‘cross-walked’ EQ-5D-3L United Kingdom (UK) value set scores. ReQoL-UI preference-based scores were obtained from its UK value set as applied to seven ReQoL-10 items. EQ-5D-5L and ReQoL measures’ construct validity and responsiveness were assessed compared against PHQ-9 and GAD-7 scores and group cut-offs.

**Results:**

361 people were randomised to intervention (241) or control (120). ReQoL-UI/-10 had better construct validity with depression severity than the EQ-5D-5L (VSE/cross-walk scores), which had relatively better construct validity with anxiety severity than the ReQoL-UI/-10. Across all intervention-arm time-points relative to baseline, responsiveness was generally better for EQ-5D-5L (VSE in particular) than ReQoL-UI, but worse than ReQoL-10.

**Conclusion:**

There is insufficient evidence to recommend the ReQoL-UI over EQ-5D-5L for economic evaluations to capture anxiety severity. However, there may be rationale for recommending the ReQoL-UI over the EQ-5D-5L to capture depression severity given its better construct validity, albeit poorer responsiveness, and if recovery-focussed quality of life relative to condition-specific symptomology is the construct of interest.

**Supplementary Information:**

The online version contains supplementary material available at 10.1007/s11136-021-02833-1.

## Plain English summary

Anxiety and depression disorders are ‘common’ mental health disorders, due to the high proportion of people they inflict. Condition-specific measures reported by those with these disorders exist to reflect condition-specific symptoms and severity. Alternatively, more ‘generic’ measures aim to capture broader aspects of physical and/or mental health in a single measure. Additionally, for allocating finite budgets between alternative care interventions, measure scores that reflect ‘preference’ between alternative health states are recommended. Our study explored to what extent two preference-based measures captured condition-specific aspects of anxiety and depression: the EQ-5D-5L is a commonly used generic measure which focusses more of physical than mental health, whereas the ReQoL-UI is a newer ‘recovery-focussed quality-of-life’ measure which focusses more of mental than physical health. Our findings suggest the commonly used EQ-5D-5L has benefits for capturing anxiety severity and was responsive as condition severity changed overtime, but the ReQoL-UI could be recommended over the EQ-5D-5L to better capture depression severity and if ‘recovery-focussed quality of life’ was of interest relative to condition-specific symptoms and severity. Overall, our findings suggest each measure has their roles for capturing aspects of anxiety and depression severity, but neither on their own captured the whole broad nature of anxiety and depressive disorders.

## Introduction

The 2010 Global Burden of Disease study estimates depression and anxiety disorders contribute a large portion of total disability amongst all mental health and substance use disorders, with increased societal costs through higher healthcare utilisation and absenteeism from work [1–4]. Mental health disorders have been estimated to represent 23% of the total cause of disability, higher than cancer and coronary heart disease [5]. In England, approximately 1/6 adults have a common mental disorder [6]. In the UK, prevalence of depressive and anxiety symptoms are significantly higher relative to pre-COVID-19 pandemic levels [7]. Therefore, prioritising mental health alongside other care interventions are important considerations for decision-makers.

Economic evaluation evidence helps inform resource allocation between alternative care interventions within a finite budget [8, 9]. Estimating the cost-effectiveness of mental health interventions has become an area of debate [8–10]. One aspect is the empirically demonstrated insensitivities to mental health aspects of health-related quality of life (HRQoL) of generic health measures compared to condition-specific measures [9–12]. This includes EuroQol’s internationally used, preference-based EQ-5D three-level version (EQ-5D-3L) used for cost-utility analysis (CUA) and recommended by reimbursement agencies including the National Institute for Health and Care Excellence (NICE) for England and Wales [13, 14]. In CUA, HRQoL measured on a preference-based scale anchored at 1 (*full health*) and 0 (*a state equivalent to dead*) is combined with length of life to generate quality-adjusted life years (QALYs), allowing comparisons between interventions that affect quantity and/or quality of life. Results suggesting the appropriateness of generic measures in patients with mental health conditions are mixed, with better support in common (e.g. anxiety and depression) relative to severe (e.g. schizophrenia and bipolar disorder) mental health populations [11, 15–17]. Therefore, it has been argued that preference-based measures focussed on the impact of the mental disorder should be considered over generic measures which often focus more on physical than mental health [17, 18].

In response to the insensitivities of the EQ-5D-3L representing 243 (3-levels^5-items^) possible health states, the EQ-5D five-level version (EQ-5D-5L) has been developed representing 3125 (5^5^) possible health states, with improved sensitivity and reduced ceiling effects [19–27]. Country-specific EQ-5D-5L preference-based value sets are available (https://euroqol.org/) with the current value set for England (VSE) based on a combined composite Time Trade-Off (cTTO) and Discrete Choice Experiment (DCE) hybrid model for eliciting preferences [28–34]. However, an independent quality assurance study raised concerns about the VSE, with NICE’s interim position being to instead use the cross-walk algorithm by van Hout et al. [35–40]. Therefore, EQ-5D-5L preference-based values can be calculated using ‘cross-walked’/‘mapped’ EQ-5D-3L United Kingdom (UK) value set scores based on the conventional TTO method [41]; however, cross-walk algorithms also have inherent concerns (e.g. predictive errors) [42–45]. EuroQol’s Blog provides updates for the new UK EQ-5D-5L valuation study [46].

The Recovering Quality-of-Life 20-item (ReQoL-20) and 10-item (ReQoL-10) version have been developed as ‘recovery-focussed quality-of-life’ measures for mental health service users [18]. A UK preference-based value set using the cTTO method can be assigned to seven ReQoL-10 items: the ReQoL Utility Index (ReQoL-UI) representing 78,125 (5^7^) possible health states as an alternative to the EQ-5D-5L for calculating QALYs in mental health service users [47]. The ReQoL-UI’s developers suggest that compared to the generic EQ-5D measures, it is a generic preference-based measure focussed more on mental than physical health [47]. Initial ReQoL-10 and ReQoL-20 psychometric analyses in a general and patient population (≈ 35%, common mental health problem) supported their internal consistency, test–retest reliability, construct validity, and responsiveness, concluding they performed “markedly better than the EQ-5D[-3L]” [18]; however, such ReQoL-UI evidence does not currently exist.

Our aim is to assess the psychometric properties (construct validity and responsiveness) of the preference-based EQ-5D-5L (VSE and cross-walk) and ReQoL-UI, compared to clinical measures for depression and anxiety: the Patient Health Questionnaire-9 (PHQ-9) and Generalised Anxiety Disorder-7 (GAD-7), respectively. Secondary psychometric analyses include the: (1) ReQoL-10, to compare its psychometric properties relative to the preference-based measures; (2) EQ-5D-5L’s single mental health ‘anxiety/depression’ item, to assess its psychometric properties for depression relative to anxiety severity given limited current evidence [48, 49].

## Methods

### Data source

Data were obtained from a parallel groups, randomised wait-list-controlled trial examining the effectiveness and cost-effectiveness of internet-delivered Cognitive Behavioural Therapy (iCBT) for depression and anxiety [50].

The study was conducted in step 2 of the Improving Access to Psychological Therapies (IAPT) program at the Berkshire National Health Service (NHS) Talking Therapies Trust. Patients are referred to IAPT Step 2 if they are experiencing mild to moderate symptoms of depression or anxiety, at which point they are offered low-intensity psychological interventions (e.g. computerised CBT) [51]. Trial participants were new IAPT referrals (June 2017–April 2018). Eligibility criteria was applied before 2:1 (intervention: 8-week waiting-list control) randomisation (Appendix S1). Trial inclusion criteria were people: (i) aged between 18 and 80 years; (ii) above clinical thresholds for depression (PHQ-9 ≥ 10) or anxiety (GAD-7 ≥ 8) [52–54], and (iii) suitable for iCBT (i.e. willing to use iCBT, internet access). In addition, the structured Mini International Neuropsychiatric Interview 7.0.2 (M.I.N.I.) [55], administered by telephone by Psychological Wellbeing Practitioners (i.e. clinicians trained in the delivery of low-intensity support) established the presence or absence of a primary diagnosis of depression or anxiety disorder at baseline.

Upon showing interest in the study, participants received an email with the information about the trial and a link to give consent through their digital signature before scheduling their M.I.N.I. Trial ethics approval was provided by the NHS England Research Ethics Committee (REC Reference: 17/NW/0311). The trial was prospectively registered: Current Controlled Trials ISRCTN91967124. Current trial status is ‘completed’ with the protocol [50] and main results [56] published. The trial results showed that iCBT produced statistically significant improvements in depression (PHQ-9) and anxiety (GAD-7) symptomatology compared to wait-list controls at 8-weeks, with further statistically significant improvements from 8-weeks up to 12-months for the intervention group [56].

### Outcome measures

Table 1 provides an overview description of the patient-reported outcome measure (PROM) scores and constructs included for psychometric analysis. Measures were collected at baseline and 8 weeks post baseline in both trial-arms; in the intervention-arm, data were also collected at 3, 6, 9, and 12 months post baseline.

**Table 1:** Description of outcomes measures and associated scores

Long name	Short name	Construct	Scoring type	No. items	Item score	Floor/ worst	Ceiling/best	Cut-offs and description	Refs.
EQ-5D five-level version	EQ-5D-5L	Preference-based generic health [depression/anxiety]	VSE (Cross-walked) [Item score]	5	5-point scale: 1 (no problem) to 5 (extreme/unable)	− 0.285 (− 0.594) [5]	1 (1) [1]	N/A (N/A) [N/A]	[25, 30, 38]
Recovering Quality of Life—Utility Index	ReQoL-UI	Preference-based recovery-focussed quality of life in mental health service users	UK value set	7	5-point scale: 0 (worst state) to 4 (best state)	− 0.195	1	N/A	[18, 47]
Recovering Quality of Life—10 item	ReQoL-10	Recovery-focussed quality of life in mental health service users	Summary	10	5-point scale: 0 (worst state) to 4 (best state)	0	40	< 24, clinical range; ≥ 24, general population	[18]
Patient Health Questionnaire-9	PHQ-9	Depression severity	Summary	9	4-point scale: 0 (not at all) to 3 (nearly every day)	27	0	< 10, No caseness; ≥ 10, Caseness < 5, Minimal; 5–9, Mild; 10–14, Moderate; 15–19, Moderately severe; ≥ 20, Severe	[54, 65]
Generalised Anxiety Disorder-7	GAD-7	Anxiety severity	Summary	7	4-point scale: 0 (not at all) to 3 (nearly every day)	21	0	< 8, No caseness; ≥ 8, Caseness < 5, Minimal; 5–9, Mild; 10–14, Moderate; ≥ 15, Severe	[52, 53, 65]

*VSE* value set for England

#### Generic measures of physical and/or mental health

The EQ-5D-5L is a self-reported, generic health measure with five severity levels scored from 1 (best state) to 5 (worst state), over five dimensions/items: mobility; self-care; usual activity; pain/discomfort; anxiety/depression [25, 57]. The VSE and cross-walk ranges from − 0.285 or − 0.594, respectively, to 1 [30, 38].

The ReQoL-10 consists of six positively worded (items: 2, 4, 5, 7, 8, 10) and four negatively worded (items: 1, 3, 6, 9) mental health items plus one physical health item to measure self-reported perception of own recovery-focussed quality of life across seven themes: autonomy; wellbeing; hope; activity; belonging and relationships; self-perception; physical health [18]. These themes align with the concept of ‘personal recovery’ and are based on outcomes mental health service users identified as being most central to them in recovering their quality of life; i.e. compared to recovery via reducing symptomatology as captured by the GAD-7 and PHQ-9 (see ‘Condition-specific measures’) [18, 58, 59]. Each ReQoL-10 item is scored from 0 (worst state) to 4 (best state), with a summary score from 0 (poorest quality of life) to 40 (highest quality of life) [18].

The ReQoL-UI can be assigned to six-items (items: 3, 5, 6, 7, 9, 10) and one physical health item of the ReQoL-10, while retaining the original seven themes [47]. The UK value set score ranges from − 0.195 to 1 [47].

#### Condition-specific measures

The PHQ-9 [54, 60] is a self-reported screening for depression measure reflecting the Diagnostic and Statistical Manual of Mental Disorders, Fourth Edition—Text Revision (DSM–IV–TR) criteria [61]. Items are scored from 0 (best state) to 3 (worst state), with a summary score range from 0 (minimal depression) to 27 (severe depression). A PHQ-9 ≥ 10 has been shown to have a sensitivity of 88% and a specificity of 88% for major depression, with five established depression severity cut-off points: “PHQ-9 scores of 5, 10, 15, and 20 represent valid and easy-to-remember thresholds demarcating the lower limits of mild, moderate, moderately severe, and severe depression” ([54], p. 611).

The GAD-7 [53] is a self-reported symptoms and severity of anxiety measure based on the DSM-IV GAD diagnostic criteria, with good internal consistency (*α* = .92) and convergent validity with other anxiety scales [53]. Items are scored from 0 (best state) to 3 (worst state), with a summary score range from 0 (minimal anxiety) to 21 (severe anxiety). A GAD-7 ≥ 10 was originally suggested to represent a reasonable cut-off for identifying GAD [53], but more recent studies have suggested using GAD-7 ≥ 8 [52, 62, 63]. The GAD-7 has four anxiety severity cut-off points: “Cut points of 5, 10, and 15 might be interpreted as representing mild, moderate, and severe levels of anxiety on the GAD-7, similar to levels of depression on the PHQ-9” ([53], p. 1095).

IAPT services have operationalised the aforementioned based on ‘caseness’ (PHQ-9 ≥ 10; GAD-7 ≥ 8) and ‘reliable change’ (PHQ-9 absolute change ≥ 6; GAD-7 absolute change ≥ 4) threshold values [52, 54, 64, 65]. Caseness is the term used to describe a patient whose symptoms of anxiety or depression are severe enough to be considered a clinical case of that condition, whereas reliable change is a change between two scores on the same measure that would be regarded as a clinically significant change in the patient’s condition [65]. These thresholds are part of IAPT’s patient-based performance outcomes when measuring ‘recovery’ (moving from ‘caseness’ to ‘no caseness’), ‘reliable improvement’ (achieving ‘reliable change’ over a course of treatment), and ‘reliable recovery’ (achieving both ‘recovery’ and ‘reliable improvement’) [65].

IAPT Phobia Scales (IAPT-PS) and Work and Social Adjustment Scale (WSAS) as routinely collected measures within IAPT services were included for analysis; these additional measures, analyses and associated results are described in the Supplementary Appendix [66–68].

### Statistical analyses

The analyses use all observed cases; therefore, the sample size (*N*) varies dependent on the analysis being performed with relevant *N* values presented in the result tables. Construct validity is assessed based on the whole cohort’s baseline data, whereas responsiveness is assessed within trial-arm across all available time-points. Statistical significance (SS), *p* < 0.05, with all analyses conducted in Stata 15 [69].

#### Construct validity

Construct validity assesses the extent to which a measure reflects HRQoL differences hypothesised to exist. This is important in relation to preference-based PROMs used to elicit QALYs, as their values should reflect HRQoL factors linked to the condition/treatment being evaluated. Construct validity is assessed despite no ‘gold standard’ HRQoL measure in mental health, given the difficulty in generating indicators that assess the full impact of mental health on people’s lives. Therefore, we assess a range of indicators to suggest, but cannot fully prove, construct validity in relation to convergent and known-group validity.

Convergent validity assesses the relationship between measures, based here on correlation analysis and locally weighted scatterplot smoothing (LOWESS) techniques. Spearman’s rank absolute correlation strength (ACS) coefficient and associated p-value as a non-parametric test, chosen post hoc based on the measures’ score distributions, indicates the degree to which instruments are measuring related factors [70]. LOWESS complements the correlation analyses as a form of non-parametric regression which plots a line of central tendency between two variables on a scatterplot, thereby visualising their general relationship across the possible score ranges without making assumptions about the actual relationship [71].

Known-group validity assesses the extent to which instrument scores differ between groups that are expected to differ, measured using Cohen’s *d* standardised absolute effect sizes (AES i.e. the difference in mean scores between two adjacent severity subgroups divided by the standard deviation of scores for the milder of the two subgroups) [70, 72]. The non-parametric Kruskal Wallis test complements assessing AES as it suggests if there is a statistically significant difference between the two or more known-groups.

#### Responsiveness

Responsiveness is important in economic evaluation as any change in health must be reflected by change in utility/preferences, and subsequent change in QALYs. For example, if HRQoL changes following an intervention, but the preference-based score does not change, this change will not be reflected in QALYs which could wrongly influence funding decisions.

To measure responsiveness we examined floor (worst possible score) and ceiling (best possible score) effects, which affect the ability of the measure to detect deterioration or improvements in health, respectively. We also examined the magnitude of change in scores over time as a crude indicator of responsiveness; however, we cross-referenced change in measure scores against the GAD-7 or PHQ-9 when reliable change had been achieved (PHQ-9 ≥ 6; GAD-7 ≥ 4) or not [67]. The assumption here is that if a change over time is observed on the condition-specific measures (general change or less/greater than the reliable change threshold), ideally we would want to observe a similar magnitude of change on the other measure scores whereby magnitude of change was assessed using standardised response mean (SRM i.e. divide the mean change on the measure by the standard deviation of the change) [70, 72].

## Results

### Descriptive statistics

Overall, 361 people were randomised (241 intervention-arm: 120 control-arm). The majority of participants were female (71.5%), ‘White/White British and Irish’ ethnicity (84.2%), employed full-time (74.5%), not prescribed psychiatric medication (51.5%), and not receiving statutory sick pay (93.4%). The M.I.N.I. classified 80.3% as having major depressive (52.4%) or anxiety disorder (64.0%), with 36.0% having both.

Table 2 presents baseline number of responders and PROM scores across the whole cohort and by trial-arm across all time-points in Table 3. At baseline, the PHQ-9 and GAD-7 suffered no missing data with the EQ-5D-5L and ReQoL-10 being completed by 355 (98.3%) and 353 (97.8%) participants, respectively. At follow-up time-points, the number of completed PROMs declined due to ‘lost to follow-up’ or ‘excluded’ from the study, or ‘unknown’ (mainly for EQ-5D-5L and ReQoL-10). As part of the trial-based analyses, data missing at follow-up was classified as missing at random [56].

**Table 2:** Outcome measure scores, floor and ceiling effects at baseline across trial-arms

Short name	*N* (%)	Mean	Median	SD	P. floor/ worst score	P. ceiling/ best score	O worst score	O best score	N floor/ worst score (%)	N ceiling/ best score (%)
PHQ-9	361 (100)	14.332	14	4.991	27	0	27	2	1 (0.3)	0 (0)
GAD-7	361 (100)	12.623	13	4.521	21	0	21	0	10 (2.8)	1 (0.3)
EQ-5D-5L VSE	355 (98.3)	0.730	0.783	0.163	− 0.285	1	− 0.010	1	0 (0)	3 (0.8)
EQ-5D-5L cross-walk	355 (98.3)	0.652	0.721	0.202	− 0.594	1	0.076	1	0 (0)	3 (0.8)
ReQoL-UI	353 (97.8)	0.778	0.807	0.141	− 0.195	1	0.115	0.995	0 (0)	0 (0)
ReQoL-10	353 (97.8)	18.598	18	6.401	0	40	3	37	0 (0)	0 (0)
EQ-5D-5L depression/anxiety	355 (98.3)	3.259	3	0.827	5	1	5	1	26 (7.3)	3 (0.8)

The table shows the possible (P.) floor/worst and ceiling/best scores as well as the observed (O.) worst and best scores achieved by the respondents; these are shown rather than possible and observed minimum and maximum scores due to the fact that for the preference-based scores a higher score is a better state, whereas for the condition-specific scores the opposite is true (i.e. a higher score is a worst state)

*EQ-5D-5L* EQ-5D Five Level version, *GAD-7* generalised anxiety disorder-7, *N* number of responder, *O.* observed, *PHQ-9* Patient Health Questionnaire-9, *P.* possible, *ReQoL-UI(-10)* Recovering Quality of Life—Utility Index (10 item), *SD* standard deviation

**Table 3:** Observed PROM scores, number of responders, and standardised response means by trial-arm and time-points

Measure	*t*_*i*_	Intervention (I), *N* = 241								Control (C), *N* = 120				
		Time-point (*t*_*i*_)		Dif. time-points, *t*_*i*_ – *t*_0_			Dif. time-points, *t*_*i*_ – *t*_*i*-1_			Time-point (*t*_*i*_)		Dif. time-points, *t*_*i*_ – *t*_0_		
		*N* (%)	Mean (SD)	*N* (%)	Mean (SD)	SRM	*N* (%)	Mean (SD)	SRM	*N* (%)	Mean (SD)	*N* (%)	Mean (SD)	SRM
PHQ-9	*t*_0_	241 (100)	14.41 (4.94)	–	–	–	–	–	–	120 (100)	14.18 (5.12)	–	–	–
	*t*_1_	198 (82)	9.28 (5.94)	198 (82)	− 5.21 (5.23)	− 0.997	198 (82)	− 5.21 (5.23)	− 0.997	91 (76)	11.58 (5.68)	91 (76)	− 2.28 (5.13)	− 0.444
	*t*_2_	186 (77)	8.17 (5.70)	186 (77)	− 6.34 (5.43)	− 1.168	177 (73)	− 0.92 (4.26)	− 0.216	–	–	–	–	–
	*t*_3_	182 (76)	7.17 (5.83)	182 (76)	− 7.12 (5.90)	− 1.206	169 (70)	− 0.82 (5.20)	− 0.157	–	–	–	–	–
	*t*_4_	177 (73)	6.81 (5.71)	177 (73)	− 7.49 (5.94)	− 1.261	167 (69)	− 0.42 (4.88)	− 0.086	–	–	–	–	–
	*t*_5_	173 (72)	6.79 (5.54)	173 (72)	− 7.56 (6.38)	− 1.184	161 (67)	− 0.10 (4.72)	− 0.021	–	–	–	–	–
GAD-7	*t*_0_	241 (100)	12.66 (4.69)	–	–	–	–	–	–	120 (100)	12.54 (4.18)	–	–	–
	*t*_1_	198 (82)	8.20 (5.31)	198 (82)	− 4.50 (5.17)	− 0.870	198 (82)	− 4.50 (5.17)	− 0.870	91 (76)	10.79 (5.12)	91 (76)	− 1.63 (4.71)	− 0.345
	*t*_2_	186 (77)	7.38 (5.32)	186 (77)	− 5.20 (5.37)	− 0.968	177 (73)	− 0.58 (4.04)	− 0.144	–	–	–	–	–
	*t*_3_	182 (76)	6.93 (5.52)	182 (76)	− 5.48 (5.98)	− 0.916	169 (70)	− 0.28 (4.85)	− 0.057	–	–	–	–	–
	*t*_4_	176 (73)	6.48 (5.14)	176 (73)	− 5.95 (5.87)	− 1.013	166 (69)	− 0.35 (4.60)	− 0.076	–	–	–	–	–
	*t*_5_	173 (72)	6.08 (4.81)	173 (72)	− 6.56 (5.87)	− 1.116	160 (66)	− 0.65 (4.35)	− 0.150	–	–	–	–	–
EQ-5D-5L	*t*_0_	238 (99)	0.735 (0.152)	–	–	–	–	–	–	117 (98)	0.722 (0.182)	–	–	–
VSE	*t*_1_	198 (82)	0.794 (0.147)	196 (81)	0.058 (0.133)	0.435	196 (81)	0.058 (0.133)	0.435	91 (76)	0.756 (0.181)	89 (74)	0.029 (0.138)	0.212
	*t*_2_	186 (77)	0.816 (0.149)	184 (76)	0.078 (0.143)	0.547	177 (73)	0.020 (0.106)	0.187	–	–	–	–	–
	*t*_3_	182 (76)	0.830 (0.171)	180 (75)	0.092 (0.168)	0.544	169 (70)	0.013 (0.141)	0.095	–	–	–	–	–
	*t*_4_	176 (73)	0.837 (0.170)	174 (72)	0.096 (0.151)	0.631	166 (69)	0.002 (0.132)	0.019	–	–	–	–	–
	*t*_5_	172 (71)	0.814 (0.172)	170 (71)	0.075 (0.167)	0.451	159 (66)	− 0.007 (0.159)	− 0.044	–	–	–	–	–
EQ-5D-5L	*t*_0_	238 (99)	0.656 (0.193)	–	–	–	–	–	–	117 (98)	0.645 (0.218)	–	–	–
cross-walk	*t*_1_	198 (82)	0.723 (0.182)	196 (81)	0.065 (0.178)	0.366	196 (81)	0.065 (0.178)	0.366	91 (76)	0.676 (0.231)	89 (74)	0.020 (0.172)	0.118
	*t*_2_	186 (77)	0.753 (0.180)	184 (76)	0.090 (0.182)	0.496	177 (73)	0.023 (0.136)	0.171	–	–	–	–	–
	*t*_3_	182 (76)	0.767 (0.212)	180 (75)	0.105 (0.217)	0.483	169 (70)	0.019 (0.178)	0.104	–	–	–	–	–
	*t*_4_	176 (73)	0.779 (0.204)	174 (72)	0.112 (0.196)	0.573	166 (69)	0.007 (0.165)	0.040	–	–	–	–	–
	*t*_5_	172 (71)	0.751 (0.201)	170 (71)	0.092 (0.215)	0.430	159 (66)	− 0.009 (0.193)	− 0.046	–	–	–	–	–
ReQoL-UI	*t*_0_	237 (98)	0.788 (0.123)	–	–	–	–	–	–	116 (97)	0.757 (0.171)	–	–	–
	*t*_1_	198 (82)	0.810 (0.140)	195 (81)	0.020 (0.141)	0.138	195 (81)	0.020 (0.141)	0.138	91 (76)	0.793 (0.163)	88 (73)	0.025 (0.139)	0.181
	*t*_2_	186 (77)	0.836 (0.151)	183 (76)	0.045 (0.152)	0.295	177 (73)	0.022 (0.139)	0.162	–	–	–	–	–
	*t*_3_	182 (76)	0.840 (0.144)	179 (74)	0.050 (0.166)	0.303	169 (70)	0.002 (0.145)	0.015	–	–	–	–	–
	*t*_4_	176 (73)	0.863 (0.124)	173 (72)	0.070 (0.117)	0.599	166 (69)	0.019 (0.142)	0.131	–	–	–	–	–
	*t*_5_	172 (71)	0.850 (0.135)	169 (70)	0.065 (0.138)	0.471	159 (66)	− 0.006 (0.127)	− 0.049	–	–	–	–	–
ReQoL-10	*t*_0_	237 (98)	18.52 (6.24)	–	–	–	–	–	–	116 (97)	18.76 (6.75)	–	–	–
	*t*_1_	198 (82)	21.00 (6.85)	195 (81)	2.20 (6.17)	0.357	195 (81)	2.20 (6.17)	0.357	91 (76)	20.25 (6.40)	88 (73)	0.93 (6.17)	0.151
	*t*_2_	186 (77)	24.10 (7.36)	183 (76)	5.37 (7.43)	0.723	177 (73)	3.04 (6.46)	0.471	–	–	–	–	–
	*t*_3_	182 (76)	24.59 (7.90)	179 (74)	5.79 (7.95)	0.728	169 (70)	0.27 (7.52)	0.036	–	–	–	–	–
	*t*_4_	176 (73)	25.49 (8.20)	173 (72)	6.66 (7.71)	0.863	166 (69)	0.92 (6.88)	0.134	–	–	–	–	–
	*t*_5_	172 (71)	24.54 (8.31)	169 (70)	6.00 (7.50)	0.801	159 (66)	− 0.66 (7.35)	− 0.090	–	–	–	–	–
EQ-5D-5L	*t*_0_	238 (99)	3.27 (0.83)	–	–	–	–	–	–	117 (98)	3.23 (0.82)	–	–	–
depression	*t*_1_	198 (82)	2.64 (0.94)	196 (81)	− 0.62 (0.95)	− 0.649	196 (81)	− 0.62 (0.95)	− 0.649	91 (76)	2.91 (1.01)	89 (74)	− 0.29 (0.87)	− 0.336
/anxiety	*t*_2_	186 (77)	2.44 (0.87)	184 (76)	− 0.78 (0.99)	− 0.784	177 (73)	− 0.16 (0.84)	− 0.195	–	–	–	–	–
item	*t*_3_	182 (76)	2.30 (0.96)	180 (75)	− 0.96 (1.08)	− 0.883	169 (70)	− 0.15 (0.97)	− 0.159	–	–	–	–	–
	*t*_4_	176 (73)	2.23 (0.99)	174 (72)	− 1.02 (1.06)	− 0.958	166 (69)	− 0.08 (0.91)	− 0.086	–	–	–	–	–
	*t*_5_	172 (71)	2.31 (0.98)	170 (71)	− 0.95 (1.06)	− 0.897	159 (66)	0.04 (0.90)	0.049	–	–	–	–	–

*t* = time point, whereby: *t*_0_ = baseline; *t*_1_ = 8 weeks; *t*_2_ = 3 months; *t*_3_ = 6 months; *t*_4_ = 9 months; *t*_5_ = 12 months

*N*(%) states the number of people who completed the measure at the specific time-point, or at two given time-points e.g. relative to *t*_0_ (baseline) or *t*_i_ (whereby *i* is any time-point denoted as 1–5)

Cohen’s SRM cut-off: < 0.2, trivial; 0.2 < 0.5, small; 0.5 < 0.8, medium; ≥ 0.8, large; an SRM of > 1 means the change in score between time-points is larger than one standard deviation

*EQ-5D-5L* EQ-5D Five Level version, *GAD-7* generalised anxiety disorder-7, *N* number of people, *ReQoL-UI(-10)* Recovering Quality of Life—Utility Index (10 item), *PHQ-9* Patient Health Questionnaire-9, *SD* standard deviation, *SRM* standardised response mean

A Consort diagram, further demographic details, histograms and additional score statistics are provided in Appendices S1–4.

### Construct validity

Table 4 ACS results and LOWESS graphs (Appendix S5) suggest that the ReQoL-UI/-10 have stronger convergent validity with depression than anxiety severity; stronger than the convergent validity results for the EQ-5D-5L scores with depression but not anxiety severity. The EQ-5D-5L scores have similar convergent validity with depression and anxiety severity.

**Table 4:** Correlation coefficient matrix between measure scores at baseline

Measures	Spearman’s rank correlation coefficient (*p*-value)				
	EQ-5D-5L VSE	EQ-5D-5L cross-walk	ReQoL-UI	ReQoL-10	EQ-5D-5L depression/anxiety
Condition-specific					
PHQ-9	− 0.391 (< 0.001)	− 0.382 (< 0.001)	− 0.529 (< 0.001)	− 0.576 (< 0.001)	0.346 (< 0.001)
GAD-7	− 0.408 (< 0.001)	− 0.411 (< 0.001)	− 0.339 (< 0.001)	− 0.331 (< 0.001)	0.514 (< 0.001)
Recovery-focussed					
ReQoL-UI	0.601 (< 0.001)	0.597 (< 0.001)	–	–	− 0.394 (< 0.001)
ReQoL-10	0.435 (< 0.001)	0.434 (< 0.001)	0.818 (< 0.001)	–	− 0.431 (< 0.001)

*EQ-5D-5L* EQ-5D Five-Level version, *GAD-7* Generalised Anxiety Disorder-7, *PHQ-9* Patient Health Questionnaire-9, *ReQoL-UI(-10)* Recovering Quality of Life—Utility Index (10 item)

Cohen’s ACS cut-offs: weak, < 0.3; moderate, 0.3 < 0.5; strong, ≥ 0.5

Table 5 AES results for caseness cut-offs suggest that the ReQoL-UI/-10 were better at quantifying a difference between depression than anxiety caseness, which the EQ-5D-5L scores did better than the ReQoL-UI/-10 for anxiety caseness, but still with a small AES. The results for the EQ-5D-5L depression/anxiety item suggests the item is better at quantifying a difference between those with anxiety than depression caseness.

**Table 5:** Testing known-group validity based on condition-specific cut-off groups at baseline

Measures	Groups, score range	*N* (%)	EQ-5D-5L VSE		EQ-5D-5L cross-walk		ReQoL-UI		ReQoL-10		EQ-5D-5L depression/anxiety	
			Mean (SD)	ES (*p*-value)	Mean (SD)	ES (*p*-value)	Mean (SD)	ES (*p*-value)	Mean (SD)	ES (*p*-value)	Mean (SD)	ES (*p*-value)
*Condition-specific*												
PHQ-9	No Caseness, < 10	65 (18.0)	0.785 (0.164)		0.712 (0.202)		0.854 (0.099)		24.219 (6.632)		3.092 (0.843)	
	Caseness, ≥ 10	296 (82.0)	0.718 (0.160)	0.413	0.639 (0.200)	0.364	0.761 (0.143)	0.688	17.353 (5.646)	1.177	3.297 (0.820)	− 0.248
				(< 0.001)		(< 0.001)		(< 0.001)		(< 0.001)		(0.052)
	Minimal, < 5	9 (2.5)	0.847 (0.095)		0.791 (0.107)		0.935 (0.036)		31.625 (4.274)		3.000 (0.707)	
	Mild, 5–9	56 (15.5)	0.775 (0.171)	0.440	0.699 (0.211)	0.455	0.843 (0.100)	0.970	23.161 (6.240)	1.399	3.107 (0.867)	− 0.126
	Moderate, 10–14	117 (32.4)	0.786 (0.115)	− 0.082	0.723 (0.140)	− 0.146	0.821 (0.089)	0.230	20.104 (4.903)	0.569	2.966 (0.658)	0.193
	Mod. Sev., 15–19	120 (33.2)	0.710 (0.164)	0.535	0.629 (0.203)	0.541	0.751 (0.138)	0.608	16.739 (5.198)	0.666	3.328 (0.832)	− 0.483
	Severe, ≥ 20	59 (16.3)	0.599 (0.159)	0.684	0.490 (0.205)	0.685	0.661 (0.177)	0.594	13.186 (4.950)	0.694	3.897 (0.742)	− 0.708
				(< 0.001)		(< 0.001)		(< 0.001)		(< 0.001)		(< 0.001)
GAD-7	No Caseness, < 8	51 (14.1)	0.787 (0.126)		0.726 (0.157)		0.823 (0.077)		20.160 (5.811)		2.706 (0.807)	
	Caseness, ≥ 8	310 (85.9)	0.721 (0.166)	0.409	0.640 (0.206)	0.431	0.770 (0.148)	0.377	18.340 (6.465)	0.285	3.352 (0.795)	− 0.811
				(0.020)		(0.016)		(0.035)		(0.044)		(< 0.001)
	Minimal, < 5	11 (3.0)	0.815 (0.124)		0.764 (0.147)		0.860 (0.064)		21.182 (5.269)		2.455 (0.688)	
	Mild, 5–9	81 (22.4)	0.794 (0.136)	0.156	0.728 (0.179)	0.207	0.826 (0.093)	0.378	21.438 (6.566)	− 0.040	2.815 (0.792)	− 0.461
	Moderate, 10–14	146 (40.4)	0.752 (0.162)	0.275	0.685 (0.184)	0.232	0.794 (0.124)	0.283	18.851 (5.914)	0.420	3.182 (0.698)	− 0.500
	Severe, ≥ 15	123 (34.1)	0.654 (0.155)	0.615	0.552 (0.201)	0.693	0.719 (0.169)	0.509	16.190 (6.064)	0.445	3.725 (0.756)	− 0.749
				(< 0.001)		(< 0.001)		(< 0.001)		(< 0.001)		(< 0.001)
*Recovery-focussed quality of life*												
ReQoL-10	Clin. range, < 24	281 (79.6)	0.707 (0.167)		0.624 (0.208)		0.746 (0.141)		16.224 (4.480)		3.361 (0.826)	
	Gen. Pop., ≥ 24	72 (20.4)	0.823 (0.101)	− 0.743	0.766 (0.119)	− 0.733	0.899 (0.044)	− 1.199	27.861 (3.825)	− 2.672	2.861 (0.718)	0.620
				(< 0.001)		(< 0.001)		(< 0.001)		(< 0.001)		(< 0.001)

*Clin.* Clinical, *ES* effect size (Cohen’s *d*), *EQ-5D-5L* EQ-5D Five Dimension Five Level version, *GAD-7* Generalised Anxiety Disorder 7, *Gen. Pop.* general population, *Mod. Sev.* moderately severe, *N* number of people, *PHQ-9* Patient Health Questionnaire 9, *ReQoL-10* Recovering Quality of Life—10 item, *ReQoL-UI* Recovering Quality of Life—Utility Index, *SD* standard deviation

Cohen’s AES cut-off: trivial, < 0.2; small, 0.2 < 0.5; medium, 0.5 < 0.8; large; ≥ 0.8; an ES of > 1 means the difference between the two means is larger than one standard deviation. ESs are relative to less severe group

*p*-values: calculated from the non-parametric Kruskal Wallis test to suggest if there is a statistically significant difference between two or more known-groups based on the scores used as a complement to assessing ES

Table 5 AES results for severity cut-offs suggests the EQ-5D-5L scores were better at quantifying a difference between ‘severe’ relative to the next adjacent severity state than any other adjacent severity states on the PHQ-9 and GAD-7, and this AES tended to be larger than for the ReQoL-UI/-10 (apart from on the PHQ-9 for ReQoL-10); however, the ReQoL-UI/-10 relative to EQ-5D-5L scores had higher AES between the adjacent lesser severe states (i.e. PHQ-9, ‘moderately severe’ relative to ‘moderate’; GAD-7, ‘moderate’ relative to ‘mild’). Additionally on the PHQ-9, the EQ-5D-5L mean scores were greater for those in the ‘moderate’ than ‘mild’ state, which seems illogical and not in-line with the other measures’ scores.

Due to the small number of people classified as ‘minimal’ (PHQ-9, *N* = 9; GAD-7, *N* = 11), these results are not described but are a limitation of the analysis. Complementary construct validity analyses at the item-level and using the IAPT-PS and WSAS measures are presented in Appendices S6 and 7.

### Responsiveness

Ceiling effects at baseline (Table 2) and at all time-points by trial-arm (Appendix S4) occurred in a lower proportion of responders for the ReQoL-UI/-10 than EQ-5D-5L.

Tables 3 and 6 SRM results suggest responsiveness differed dependent on time-points being compared relative to baseline (e.g. largest SRMs at 9 months across all measures). PHQ-9 and GAD-7 responsiveness was generally large. EQ-5D-5L scores were relatively more responsive than the ReQoL-UI across all time-points assessed, but the ReQoL-10 tended to be more responsive than its preference-based counterparts.

**Table 6:** Standardised response means (SRM)—intervention-arm participants grouped dependent on reliable change in PHQ-9 or GAD-7 score since baseline

Measure	*t*_*i*_	PHQ-9	PHQ-9						GAD-7					
		GAD-7	PHQ-9 Δ ≤ − 6			PHQ-9 Δ − 6 < 6			GAD-7 Δ ≤ − 4			GAD-7 Δ − 4 < 4		
		*N* (%)	*N* (%)	Mean (SD)	SRM	*N* (%)	Mean (SD)	SRM	*N* (%)	Mean (SD)	SRM	*N* (%)	Mean (SD)	SRM
EQ-5D-5L	*t*_1_–*t*_0_	196 (81)	85 (43)	0.102 (0.138)	0.734	109 (56)	0.029 (0.116)	0.252	109 (56)	0.088 (0.136)	0.647	79 (40)	0.021 (0.122)	0.175
VSE	*t*_2_–*t*_0_	184 (76)	103 (56)	0.115 (0.134)	0.856	79 (43)	0.032 (0.141)	0.231	115 (63)	0.112 (0.131)	0.855	61 (33)	0.037 (0.141)	0.259
	*t*_3_–*t*_0_	180 (75)	109 (61)	0.136 (0.157)	0.865	68 (38)	0.035 (0.137)	0.258	114 (63)	0.129 (0.159)	0.815	54 (30)	0.064 (0.118)	0.538
	*t*_4_–*t*_0_	174 (72)	114 (66)	0.129 (0.136)	0.943	58 (33)	0.044 (0.151)	0.291	118 (68)	0.123 (0.132)	0.930	49 (28)	0.054 (0.174)	0.310
	*t*_5_–*t*_0_	170 (71)	107 (63)	0.112 (0.141)	0.794	58 (34)	0.027 (0.185)	0.145	122 (72)	0.110 (0.139)	0.792	39 (23)	0.012 (0.200)	0.061
EQ-5D-5L	*t*_1_–*t*_0_	196 (81)	85 (43)	0.118 (0.182)	0.648	109 (56)	0.031 (0.156)	0.201	109 (56)	0.104 (0.182)	0.573	79 (40)	0.017 (0.165)	0.105
cross-walk	*t*_2_–*t*_0_	184 (76)	103 (56)	0.135 (0.164)	0.823	79 (43)	0.035 (0.191)	0.183	115 (63)	0.132 (0.163)	0.808	61 (33)	0.039 (0.190)	0.205
	*t*_3_–*t*_0_	180 (75)	109 (61)	0.153 (0.208)	0.735	68 (38)	0.052 (0.174)	0.302	114 (63)	0.149 (0.203)	0.731	54 (30)	0.080 (0.174)	0.462
	*t*_4_–*t*_0_	174 (72)	114 (66)	0.150 (0.170)	0.884	58 (33)	0.052 (0.215)	0.243	118 (68)	0.144 (0.178)	0.812	49 (28)	0.070 (0.205)	0.340
	*t*_5_–*t*_0_	170 (71)	107 (63)	0.132 (0.189)	0.699	58 (34)	0.048 (0.234)	0.207	122 (72)	0.135 (0.186)	0.727	39 (23)	0.011 (0.247)	0.043
ReQoL-UI	*t*_1_–*t*_0_	195 (81)	85 (44)	0.048 (0.144)	0.334	108 (55)	− 0.001 (0.137)	− 0.009	109 (56)	0.037 (0.143)	0.262	78 (40)	− 0.006 (0.131)	− 0.049
	*t*_2_–*t*_0_	183 (76)	103 (56)	0.087 (0.134)	0.646	78 (43)	− 0.008 (0.159)	− 0.052	115 (63)	0.074 (0.145)	0.510	60 (33)	0.005 (0.145)	0.036
	*t*_3_–*t*_0_	179 (74)	109 (61)	0.085 (0.159)	0.533	67 (37)	0.006 (0.158)	0.037	113 (63)	0.087 (0.155)	0.563	54 (30)	− 0.001 (0.159)	− 0.006
	*t*_4_–*t*_0_	173 (72)	113 (65)	0.102 (0.096)	1.059	58 (34)	0.012 (0.129)	0.096	116 (67)	0.094 (0.103)	0.911	50 (29)	0.038 (0.125)	0.303
	*t*_5_–*t*_0_	169 (70)	108 (64)	0.097 (0.134)	0.729	56 (33)	0.013 (0.129)	0.103	122 (72)	0.091 (0.121)	0.748	38 (22)	0.000 (0.168)	− 0.003
ReQoL-10	*t*_1_–*t*_0_	195 (81)	85 (44)	4.506 (7.243)	0.622	108 (55)	0.500 (4.423)	0.113	109 (56)	3.385 (6.409)	0.528	78 (40)	0.590 (5.740)	0.103
	*t*_2_–*t*_0_	183 (76)	103 (56)	8.359 (7.097)	1.178	78 (43)	1.679 (5.839)	0.288	115 (63)	7.174 (7.430)	0.966	60 (33)	2.367 (6.460)	0.366
	*t*_3_–*t*_0_	179 (74)	109 (61)	8.817 (7.961)	1.107	67 (37)	1.403 (4.939)	0.284	113 (63)	8.310 (7.500)	1.108	54 (30)	2.352 (6.519)	0.361
	*t*_4_–*t*_0_	173 (72)	113 (65)	9.124 (7.558)	1.207	58 (34)	2.155 (5.448)	0.396	116 (67)	8.586 (7.752)	1.108	50 (29)	3.160 (6.089)	0.519
	*t*_5_–*t*_0_	169 (70)	108 (64)	8.361 (7.245)	1.154	56 (33)	2.214 (6.050)	0.366	122 (72)	7.705 (7.159)	1.076	38 (22)	1.868 (7.018)	0.266
EQ-5D-5L	*t*_1_–*t*_0_	196 (81)	85 (43)	− 1.000 (1.000)	− 1.000	109 (56)	− 0.349 (0.786)	− 0.443	109 (56)	− 0.881 (1.007)	− 0.875	79 (40)	− 0.304 (0.774)	− 0.393
depression	*t*_2_–*t*_0_	184 (76)	103 (56)	− 1.010 (0.923)	− 1.093	79 (43)	− 0.494 (0.998)	− 0.494	115 (63)	− 1.035 (0.936)	− 1.106	61 (33)	− 0.443 (0.922)	− 0.480
/anxiety	*t*_3_–*t*_0_	180 (75)	109 (61)	− 1.312 (0.978)	− 1.341	68 (38)	− 0.471 (0.969)	− 0.486	114 (63)	− 1.272 (0.989)	− 1.286	54 (30)	− 0.648 (0.935)	− 0.693
item	*t*_4_–*t*_0_	174 (72)	114 (66)	− 1.333 (0.899)	− 1.483	58 (33)	− 0.483 (1.047)	− 0.461	118 (68)	− 1.280 (0.995)	− 1.286	49 (28)	− 0.592 (0.956)	− 0.619
	*t*_5_–*t*_0_	170 (71)	107 (63)	− 1.215 (0.911)	− 1.333	58 (34)	− 0.621 (1.089)	− 0.570	122 (72)	− 1.230 (0.907)	− 1.355	39 (23)	− 0.359 (1.088)	− 0.330

Groupings based on what is classified as a reliable change (Δ) in score, which for the PHQ-9 is an absolute score value of ≥ 6 [67] and for the GAD-7 is an absolute score value of ≥ 4 [67]

Cohen’s SRM cut-off: < 0.2, trivial; 0.2 < 0.5, small; 0.5 < 0.8, medium; ≥ 0.8, large; an SRM of > 1 means the change in score between time-points is larger than one standard deviation

*EQ-5D-5L* EQ-5D Five Dimension Five Level version, *GAD-7* Generalised Anxiety Disorder 7, *N* number of people, *PHQ-9* Patient Health Questionnaire 9, *ReQoL-10* Recovering Quality of Life—10 item, *ReQoL-UI* Recovering Quality of Life—Utility Index, *SD* standard deviation, *SRM* standardised response mean

## Discussion

In terms of preference-based measures and scores used for economic evaluations of interventions for anxiety and depression as common comorbid conditions [73], recommending either the EQ-5D-5L (VSE or cross-walk) or ReQoL-UI to cover the severity range of both conditions does not seem clear-cut based on these results.

For capturing anxiety severity, the recommendation given our findings is to use the EQ-5D-5L rather than ReQoL-UI. The psychometric properties of the EQ-5D-3L in those with anxiety and depression has previously been assessed, the general results suggesting construct validity and responsiveness for depression, but the results for anxiety severity are less convincing [10, 16, 17, 74, 75]. When the EQ-5D-5L has been psychometrically compared against the EQ-5D-3L in study samples including those with depression and/or anxiety, the results have generally suggested the EQ-5D-5L improves on the EQ-5D-3L based on reduced ceiling effects, and improved discriminatory power (known-group validity) and convergent validity [26, 27]; however, due to the inclusion of people with multiple conditions with no anxiety or depression-specific measure and use of different statistical methods, direct comparison with these studies is difficult.

When comparing the EQ-5D-5L VSE and cross-walk in our study, they have similar psychometric results. Within the UK, Mulhern et al. [76] compared the UK EQ-5D-3L value set, EQ-5D-5L VSE and cross-walk concluding that there are important differences, including the distribution of the value sets systematically differed (e.g. Appendix S3) and the EQ-5D-5L values were higher than EQ-5D-3L/cross-walk values (e.g. Tables 2 and 3). Despite these identified differences, our psychometric results based on VSE and cross-walk in terms of construct validity were similar, with better responsiveness for the VSE relative to cross-walked scores—the suggestion being the preference-based scores may play more of a part in the measures’ responsiveness than construct validity, which logically make sense given the ‘construct’ should stem from the descriptive system but ‘responsiveness’ will be related to the scoring algorithm used.

Compared to the EQ-5D-5L, the ReQoL-UI/-10 clearly have better construct validity with depression than anxiety severity. One explanation could be that the GAD-7 is focussed on anxiety symptomology, whereas the ReQoL-UI/-10 departs from symptomology as recovery-focussed quality-of-life measures such that by construct design they wouldn’t be capturing similar aspects of anxiety; although, more symptomatic items are included in the ReQoL-20. However, responsiveness was generally small for the ReQoL-UI (medium when GAD-7/PHQ-9 > reliable change threshold); smaller than for the EQ-5D-5L scores and ReQoL-10. Direct comparisons with the psychometric assessment which suggested the ReQoL-10 performed “markedly better than the EQ-5D[-3L]” are difficult, but our results suggest the ReQoL-10 generally had better responsiveness and construct validity with depression than the EQ-5D-5L, but not with anxiety severity [18]. The ReQoL-UI’s UK value set and less mental health items compared to the ReQoL-10 seems to be reducing its relative responsiveness; however, as the first study assessing the ReQoL-UI’s psychometric properties, there are currently no comparative empirical literature results.

The EQ-5D-5L’s single mental health ‘anxiety/depression’ item captured anxiety and depression constructs differently, generally having better construct validity with anxiety than depression severity. This item-level result has been suggested by previous studies, albeit suggesting the item captures aspects/changes associated with depression better than anxiety, but certainly not equally across constructs [48, 49]. When assessing convergent validity at the item-level particularly for the preference-based measures, specific items are potentially driving the convergent validity results before accounting for the influence of the preference-based scores (e.g. EQ-5D-5L’s ‘anxiety/depression’ item with GAD-7 items and score)—see Appendix S6.

### Reimbursement and policy implications

The results of this study highlight a range of considerations when using and interpreting scores from the EQ-5D-5L and ReQoL-UI (-10), and their subsequent effect on economic evaluation (or clinical assessment) evidence. We shall focus on two implications from a reimbursement and policy perspective, particularly associated with NICE given our focus on England/UK value sets and cross-walk.

First, our results suggest the VSE has marginally better psychometric properties over the NICE recommended cross-walk for capturing the impact of anxiety and/or depression severity [37]. However, these results are perhaps not sufficient to make NICE change their interim position at this time given the ongoing debate around the VSE for which there is a new valuation study [35, 36, 39, 40, 46]. Further work is required to understand how the EQ-5D-5L (VSE and cross-walk) and ReQoL-UI impact on QALY and subsequent cost-effectiveness estimates provided to decision-makers (the current authors are assessing this aspect for a future publication).

Secondly, different preference-based measures, value sets and cross-walk algorithms produce different QALYs [77, 78], which is partly behind NICE’s EQ-5D-3L reference case to produce directly comparable results [13, 37]. However, agencies like NICE state alternative preference-based measures can be used if supported by empirical evidence, such as comparative psychometric results [13]. Here we suggest the EQ-5D-5L (VSE and cross-walk) better captures anxiety severity with better responsiveness than the ReQoL-UI. As the NICE preferred measure, there is no suggestion to choose the ReQoL-UI over the EQ-5D-5L if only one can be chosen to capture anxiety severity (this will be down to researchers, patients and public representatives to deliberate the extra cognitive burden of additional questions on the patient group of interest). For depression severity, the ReQoL-UI’s better construct validity, despite its poorer responsiveness, may be enough to rationalise its use over the EQ-5D-5L; noting depression severity will be notably better represented than anxiety severity by the ReQoL-UI and responsiveness is important particularly for economic evaluation. However, the ReQoL-10 offers both a clinical *and* preference-based measure, which could capture additional information important to patients, clinicians, and decision-makers.

Additionally, the ReQoL measures are designed to depart from symptomology to broader recovery-focussed quality of life. Although PHQ-9 and GAD-7 measures are used to capture symptomology in IAPT service users with ‘[symptom] recovery’ representing a change from ‘caseness’ to ‘no caseness’, a shift in paradigm to these broader ‘personal recovery’ aspects could change the interpretation of our results if the symptoms and severity aspects captured by the GAD-7 and PHQ-9 were no longer the outcomes of interest for mental health services and users.

### International generalisability

The generalisability of our England/UK-based results to other countries requires reflecting on existing between country considerations of value sets, cross-walk algorithms, and descriptive systems. For example, Gerlinger et al. [79] compared EQ-5D-5L value sets across six different countries (Canada, England, Japan, Korea, Netherlands, and Uruguay) and 10 different cross-walk algorithms: “There were substantial differences in the [value set] utility index between countries in the values attributed to each health state”; a suggestion also made related to cross-walked scores. It is difficult to hypothesise exactly how psychometric performance might change between country-specific value sets and cross-walk algorithms, noting that part of the psychometric properties comes from the underlying descriptive system which will remain (hopefully) intact across countries. Although translation and subsequent interpretation of the descriptive system could impact on results, it seems reasonable to suggest the construct validity results which stem more from the measures’ descriptive system may be generalisable, but the responsiveness results are more country specific dependent on value set and cross-walk algorithm used while noting the limitation of our indirect comparison [80].

## Limitations

These trial eligible participants represent a specific mental health population referred to IAPT Step 2 care, England; therefore, they do not represent the full range of anxiety and/or depression severity (i.e. few had ‘minimal’ severity, restricting analysis at this level). The sample size is greater than the ‘rules of thumb’ for the analyses conducted [81]; however, larger and more representative samples of patients with depression and anxiety, using alternative measures alongside the GAD-7 and PHQ-9 (e.g. HADS; BDI-II) for comparison and in diverse settings (e.g. secondary mental health care), should be sought. Without a gold standard, indirect methods are used to support the psychometric results. If there is a shift from ‘symptom recovery’ to broader ‘personal recovery’ within mental health services like IAPT, the ReQoL measures purport to measure this construct and therefore condition-specific, more symptom-based measures like the GAD-7 and PHQ-9 may not represent the construct of interest which form the basis on this psychometric analysis.

## Conclusion

ReQoL-UI/-10 had better construct validity with depression severity (PHQ-9) than the EQ-5D-5L (VSE and cross-walk scores), which had relatively better construct validity with anxiety severity (GAD-7) than the ReQoL-UI/-10. EQ-5D-5L score responsiveness (VSE particularly) was better than ReQoL-UI, but worse than ReQoL-10. EQ-5D-5L anxiety/depression item had better construct validity with anxiety than depression severity. There is insufficient evidence to suggest using the ReQoL-UI over EQ-5D-5L for economic evaluations to capture anxiety severity. However, there may be rationale to use the ReQoL-UI to capture depression severity given its better construct validity, albeit poorer responsiveness, and if ‘personal recovery’ relative to change in symptomology is the construct/outcome of interest for mental health services and users.

## Supplementary Information

Below is the link to the electronic supplementary material.

Supplementary file1 (DOCX 1927 kb)

## Data Availability

The dataset analysed during the current study can be made available upon request to the co-authors: AE and DR.

## Code availability

The code used for the purpose of psychometric analysis can be made available upon request to the corresponding author.

## References

[1] Evans-Lacko S, Knapp M (2016). Global patterns of workplace productivity for people with depression: Absenteeism and presenteeism costs across eight diverse countries. Social Psychiatry and Psychiatric Epidemiology.

[2] Katon WJ, Lin E, Russo J, Unützer J (2003). Increased medical costs of a population-based sample of depressed elderly patients. Archives of General Psychiatry.

[3] Simon G, Ormel J, VonKorff M, Barlow W (1995). Health care costs associated with depressive and anxiety disorders in primary care. American Journal of Psychiatry.

[4] Whiteford HA, Degenhardt L, Rehm J, Baxter AJ, Ferrari AJ, Erskine HE, Charlson FJ, Norman RE, Flaxman AD, Johns N (2013). Global burden of disease attributable to mental and substance use disorders: Findings from the Global Burden of Disease Study 2010. The Lancet.

[5] Hewlett, E., & Horner, K., (2015). Mental health analysis profiles (MhAPs): England, OECD Health Working Papers 81, OECD Publishing. https://ideas.repec.org/p/oec/elsaad/81-en.html.

[6] McManus, S., Bebbington, P., Jenkins, R., & Brugha, T. (2016). Mental health and wellbeing in England: Adult Psychiatric Morbidity Survey 2014: A survey carried out for NHS Digital by NatCen Social Research and the Department of Health Sciences, University of Leicester: NHS Digital.

[7] Pieh C, Budimir S, Delgadillo J, Barkham M, Fontaine JR, Probst T (2020). Mental health during COVID-19 lockdown in the United Kingdom. Psychosomatic Medicine.

[8] Razzouk, D. (2017). *Mental health economics: The costs and benefits of psychiatric care*. Springer.

[9] Franklin, M. (2017). Cost utility analysis. In R. D (Ed.), *Mental health economics* (pp. 89–119). Springer.

[10] Brazier J (2008). Measuring and valuing mental health for use in economic evaluation. Journal of Health Services Research & Policy.

[11] Brazier J, Connell J, Papaioannou D, Mukuria C, Mulhern B, Peasgood T, Jones ML, Paisley S, O'Cathain A, Barkham M (2014). A systematic review, psychometric analysis and qualitative assessment of generic preference-based measures of health in mental health populations and the estimation of mapping functions from widely used specific measures. Health Technology Assessment (Winchester, England).

[12] Brazier, J., Rowen, D., Mavranezouli, I., Tsuchiya, A., Young, T., Yang, Y., Barkham, M., & Ibbotson, R. (2012). Developing and testing methods for deriving preference-based measures of health from condition-specific measures (and other patient-based measures of outcome). In NIHR Health Technology Assessment programme: Executive Summaries: NIHR Journals Library. 10.3310/hta16320 22832015

[13] NICE. (2013). *Guide to the methods of technology appraisal*. National Institute for Health and Care Excellence (NICE). 27905712

[14] Rowen D, Zouraq IA, Chevrou-Severac H, van Hout B (2017). International regulations and recommendations for utility data for health technology assessment. PharmacoEconomics.

[15] Brazier J, Roberts J, Tsuchiya A, Busschbach J (2004). A comparison of the EQ-5D and SF-6D across seven patient groups. Health Economics.

[16] Payakachat N, Ali MM, Tilford JM (2015). Can the EQ-5D detect meaningful change? A systematic review. Pharmacoeconomics.

[17] Mulhern B, Mukuria C, Barkham M, Knapp M, Byford S, Brazier J (2014). Using generic preference-based measures in mental health: Psychometric validity of the EQ-5D and SF-6D. The British Journal of Psychiatry.

[18] Keetharuth AD, Brazier J, Connell J, Bjorner JB, Carlton J, Buck ET, Ricketts T, McKendrick K, Browne J, Croudace T (2018). Recovering Quality of Life (ReQoL): A new generic self-reported outcome measure for use with people experiencing mental health difficulties. The British Journal of Psychiatry.

[19] Golicki D, Niewada M, Karlińska A, Buczek J, Kobayashi A, Janssen M, Pickard AS (2015). Comparing responsiveness of the EQ-5D-5L, EQ-5D-3L and EQ VAS in stroke patients. Quality of Life Research.

[20] Buchholz I, Thielker K, Feng Y-S, Kupatz P, Kohlmann T (2015). Measuring changes in health over time using the EQ-5D 3L and 5L: A head-to-head comparison of measurement properties and sensitivity to change in a German inpatient rehabilitation sample. Quality of Life Research.

[21] Janssen MF, Birnie E, Haagsma JA, Bonsel GJ (2008). Comparing the standard EQ-5D three-level system with a five-level version. Value in Health.

[22] Pickard AS, De Leon MC, Kohlmann T, Cella D, Rosenbloom S (2007). Psychometric comparison of the standard EQ-5D to a 5 level version in cancer patients. Medical Care.

[23] Scalone L, Ciampichini R, Fagiuoli S, Gardini I, Fusco F, Gaeta L, Del Prete A, Cesana G, Mantovani LG (2013). Comparing the performance of the standard EQ-5D 3L with the new version EQ-5D 5L in patients with chronic hepatic diseases. Quality of Life Research.

[24] Golicki D, Zawodnik S, Janssen MF, Kiljan A, Hermanowski T (2010). Psychometric comparison of EQ-5D and EQ-5D-5L in student population. Value in Health.

[25] Herdman M, Gudex C, Lloyd A, Janssen M, Kind P, Parkin D, Bonsel G, Badia X (2011). Development and preliminary testing of the new five-level version of EQ-5D (EQ-5D-5L). Quality of life research.

[26] Agborsangaya CB, Lahtinen M, Cooke T, Johnson JA (2014). Comparing the EQ-5D 3L and 5L: Measurement properties and association with chronic conditions and multimorbidity in the general population. Health and Quality of Life Outcomes.

[27] Janssen M, Pickard AS, Golicki D, Gudex C, Niewada M, Scalone L, Swinburn P, Busschbach J (2013). Measurement properties of the EQ-5D-5L compared to the EQ-5D-3L across eight patient groups: A multi-country study. Quality of Life Research.

[28] Oppe M, Devlin NJ, van Hout B, Krabbe PF, de Charro F (2014). A program of methodological research to arrive at the new international EQ-5D-5L valuation protocol. Value in Health.

[29] Stolk E, Ludwig K, Rand K, van Hout B, Ramos-Goñi JM (2019). Overview, update, and lessons learned from the International EQ-5D-5L valuation work: Version 2 of the EQ-5D-5L valuation protocol. Value in Health.

[30] Devlin NJ, Shah KK, Feng Y, Mulhern B, van Hout B (2018). Valuing health-related quality of life: An EQ-5D-5L value set for England. Health Economics.

[31] Devlin NJ, Tsuchiya A, Buckingham K, Tilling C (2011). A uniform time trade off method for states better and worse than dead: Feasibility study of the ‘lead time’ approach. Health Economics.

[32] Janssen BM, Oppe M, Versteegh MM, Stolk EA (2013). Introducing the composite time trade-off: A test of feasibility and face validity. The European Journal of Health Economics.

[33] Ramos-Goñi JM, Pinto-Prades JL, Oppe M, Cabasés JM, Serrano-Aguilar P, Rivero-Arias O (2017). Valuation and modeling of EQ-5D-5L health states using a hybrid approach. Medical Care.

[34] Rowen D, Brazier J, Van Hout B (2015). A comparison of methods for converting DCE values onto the full health-dead QALY scale. Medical Decision Making.

[35] Hernández-Alava, M., Pudney, S., & Wailoo, A. (2018). Quality review of a proposed EQ-5D-5L value set for England. EEPRU report [online]. 10.1016/j.jval.2019.10.017 32389230

[36] Hernandez-Alava M, Pudney S, Wailoo A (2020). The EQ-5D-5L value set for England: Findings of a quality assurance program. Value in Health.

[37] NICE. (2018). Position statement on use of the EQ-5D-5L valuation set for England (updated November 2018). Retrieved April 16, 2019, from https://www.nice.org.uk/about/what-we-do/our-programmes/nice-guidance/technology-appraisal-guidance/eq-5d-5l

[38] van Hout B, Janssen M, Feng Y-S, Kohlmann T, Busschbach J, Golicki D, Lloyd A, Scalone L, Kind P, Pickard AS (2012). Interim scoring for the EQ-5D-5L: Mapping the EQ-5D-5L to EQ-5D-3L value sets. Value in Health.

[39] Norman R, Olsen JA (2020). Competing views on the English EQ-5D-5L valuation set. Value in Health.

[40] van Hout B, Mulhern B, Feng Y, Shah K, Devlin N (2020). The EQ-5D-5L value set for England: Response to the “Quality Assurance”. Value in Health.

[41] Dolan P (1997). Modeling valuations for EuroQol health states. Medical Care.

[42] Mukuria C, Rowen D, Harnan S, Rawdin A, Wong R, Ara R, Brazier J (2019). An updated systematic review of studies mapping (or cross-walking) measures of health-related quality of life to generic preference-based measures to generate utility values. Applied Health Economics and Health Policy.

[43] Longworth L, Rowen D (2011). NICE DSU technical support document 10: The use of mapping methods to estimate health state utility values.

[44] Hernández Alava, M., Pudney, S., & Wailoo, A. (2020). Estimating the relationship between EQ-5D-5L and EQ-5D-3L: Results from an English Population Study. University of Sheffield & University of York. 10.1007/s40273-022-01218-7 PMC9883358 36449173

[45] Hernández-Alava M, Pudney S (2018). eq5dmap: A command for mapping between EQ-5D-3L and EQ-5D-5L. The Stata Journal.

[46] EuroQol. (2020). New UK EQ-5D-5L valuation study—Blog. Retrieved March 2, 2020, from https://euroqol.org/eq-5d-instruments/eq-5d-5l-about/valuation-standard-value-sets/new-uk-eq-5d-5l-valuation-study_blog/

[47] Keetharuth AD, Rowen D, Bjorner J, Brazier J (2020). Estimating a Preference-Based Index for mental health from the Recovering Quality of Life (ReQoL) measure: Valuation of ReQoL-UI. Value in Health.

[48] Crick K, Al Sayah F, Ohinmaa A, Johnson JA (2018). Responsiveness of the anxiety/depression dimension of the 3-and 5-level versions of the EQ-5D in assessing mental health. Quality of Life Research.

[49] Supina AL, Johnson JA, Patten SB, Williams JV, Maxwell CJ (2007). The usefulness of the EQ-5D in differentiating among persons with major depressive episode and anxiety. Quality of Life Research.

[50] Richards D, Duffy D, Blackburn B, Earley C, Enrique A, Palacios J, Franklin M, Clarke G, Sollesse S, Connell S (2018). Digital IAPT: The effectiveness & cost-effectiveness of internet-delivered interventions for depression and anxiety disorders in the Improving Access to Psychological Therapies programme: Study protocol for a randomised control trial. BMC Psychiatry.

[51] Clark DM (2011). Implementing NICE guidelines for the psychological treatment of depression and anxiety disorders: The IAPT experience. International Review of Psychiatry.

[52] Kroenke K, Spitzer RL, Williams JB, Monahan PO, Löwe B (2007). Anxiety disorders in primary care: Prevalence, impairment, comorbidity, and detection. Annals of Internal Medicine.

[53] Spitzer RL, Kroenke K, Williams JB, Löwe B (2006). A brief measure for assessing generalized anxiety disorder: The GAD-7. Archives of Internal Medicine.

[54] Kroenke K, Spitzer RL, Williams JB (2001). The PHQ-9: Validity of a brief depression severity measure. Journal of General Internal Medicine.

[55] Sheehan DV, Lecrubier Y, Sheehan KH, Amorim P, Janavs J, Weiller E, Hergueta T, Baker R, Dunbar GC (1998). The Mini-International Neuropsychiatric Interview (MINI): The development and validation of a structured diagnostic psychiatric interview for DSM-IV and ICD-10. The Journal of Clinical Psychiatry.

[56] Richards D, Enrique A, Eilert N, Franklin M, Palacios J, Duffy D, Earley C, Chapman J, Jell G, Sollesse S (2020). A pragmatic randomized waitlist-controlled effectiveness and cost-effectiveness trial of digital interventions for depression and anxiety. NPJ Digital Medicine.

[57] EuroQol. (2020). EQ-5D-5L user guide. Retrieved February 1, 2021, from https://euroqol.org/publications/user-guides/

[58] Leamy M, Bird V, Le Boutillier C, Williams J, Slade M (2011). Conceptual framework for personal recovery in mental health: Systematic review and narrative synthesis. The British Journal of Psychiatry.

[59] Slade, M. (2009). Personal recovery and mental illness: A guide for mental health professionals. University Press.

[60] Spitzer, R. L., Kroenke, K., Williams, J. B., & Group, P. H. Q. P. C. S (1999). Validation and utility of a self-report version of PRIME-MD: The PHQ primary care study. JAMA.

[61] American Psychiatric Association. (2000). Diagnostic and statistical manual of mental disorders (DSM-IV-TR) (4th ed.). American Psychiatric Association (APA).

[62] Rutter LA, Brown TA (2017). Psychometric properties of the generalized anxiety disorder scale-7 (GAD-7) in outpatients with anxiety and mood disorders. Journal of Psychopathology and Behavioral Assessment.

[63] Johnson SU, Ulvenes PG, Øktedalen T, Hoffart A (2019). Psychometric properties of the general anxiety disorder 7-item (GAD-7) scale in a heterogeneous psychiatric sample. Frontiers in Psychology.

[64] Gyani A, Shafran R, Layard R, Clark DM (2013). Enhancing recovery rates: Lessons from year one of IAPT. Behaviour Research and Therapy.

[65] NHS Digital. (2021). A guide to IAPT data and publications. Retrieved February 1, 2021, from https://digital.nhs.uk/binaries/content/assets/website-assets/data-and-information/data-sets/iapt/iapt-v2.0-docs/iapt-v2.0-guidance-document.pdf

[66] NHS. (2011). *The improving access to psychological therapies data handbook v2.0.1*. N. H. S. (NHS).

[67] NHS. (2018). *The improving access to psychological therapies manual: Appendices and helpful resources, version 1*. N. H. S. (NHS).

[68] Mundt JC, Marks IM, Shear MK, Greist JM (2002). The Work and Social Adjustment Scale: A simple measure of impairment in functioning. The British Journal of Psychiatry.

[69] StataCorp. (2017). Stata Statistical Software: Release 15.

[70] Cohen J (1992). A power primer. Psychological Bulletin.

[71] Cleveland WS (1979). Robust locally weighted regression and smoothing scatterplots. Journal of the American Statistical Association.

[72] Middel, B., & van Sonderen, E. (2001). How to interpret the magnitude of change in health-related quality of life? A study on the use of Cohen’s thresholds for effect size estimates. In L. J. Middel (Ed.), *Assessment of change in clinical evaluation*. University of Groningen.

[73] Spinhoven P, van Balkom A, Nolen WA (2011). Comorbidity patterns of anxiety and depressive disorders in a large cohort study: The Netherlands Study of Depression and Anxiety (NESDA). Journal of Clinical Psychiatry.

[74] Brazier J (2010). Is the EQ–5D fit for purpose in mental health?. The British Journal of Psychiatry.

[75] Peasgood, T., Brazier, J., & Papaioannou, D. (2012). A systematic review of the validity and responsiveness of EQ-5D and SF-6D for depression and anxiety. HEDS Discussion paper 12/15.

[76] Mulhern B, Feng Y, Shah K, Janssen MF, Herdman M, van Hout B, Devlin N (2018). Comparing the UK EQ-5D-3L and English EQ-5D-5L value sets. PharmacoEconomics.

[77] Richardson J, Khan MA, Iezzi A, Maxwell A (2015). Comparing and explaining differences in the magnitude, content, and sensitivity of utilities predicted by the EQ-5D, SF-6D, HUI 3, 15D, QWB, and AQoL-8D multiattribute utility instruments. Medical Decision Making.

[78] Pickles K, Lancsar E, Seymour J, Parkin D, Donaldson C, Carter SM (2019). Accounts from developers of generic health state utility instruments explain why they produce different QALYs: A qualitative study. Social Science & Medicine.

[79] Gerlinger C, Bamber L, Leverkus F, Schwenke C, Haberland C, Schmidt G, Endrikat J (2019). Comparing the EQ-5D-5L utility index based on value sets of different countries: Impact on the interpretation of clinical study results. BMC Research Notes.

[80] Devlin NJ, Brooks R (2017). EQ-5D and the EuroQol group: Past, present and future. Applied Health Economics and Health Policy.

[81] VanVoorhis CW, Morgan BL (2007). Understanding power and rules of thumb for determining sample sizes. Tutorials in Quantitative Methods for Psychology.

