# Supplementary material for: Psychometric assessment of EQ-5D-5L and ReQoL measures in patients with anxiety and depression: construct validity and responsiveness
Source: Qual Life Res. 2021 Apr 9;30(9):2633–47. doi: 10.1007/s11136-021-02833-1 (PMC8034045; doi:10.1007/s11136-021-02833-1)
Supplement: Supplementary file 1 — Supplementary file1 (DOCX 1927 kb) [file 11136_2021_2833_MOESM1_ESM.docx]

**Full title: Psychometric assessment of EQ-5D-5L and ReQoL measures in patients with anxiety and depression: construct validity and responsiveness**

**Short title: Psychometrics of EQ-5D-5L and ReQoL measures**

**Supplementary Appendices**

**Contents Page**

[Appendix S1: Recruitment, eligibility criteria and Consort diagram 2](#_Toc65866313)

[Appendix S2: Baseline demographics and patient characteristics 3](#_Toc65866314)

[Appendix S3: Histograms of outcome measure score distributions at baseline across trial-arms 5](#_Toc65866315)

[Appendix S4: Generic measure scores including ceiling and floor effects by trial-arm and time-point 8](#_Toc65866316)

[Appendix S5: Assessing Convergent validity using locally weighted scatterplot smoothing (LOWESS) techniques 11](#_Toc65866317)

[Appendix S6: Assessing Convergent validity at the item-level – a complementary analysis 17](#_Toc65866318)

[Appendix S7: Beyond the GAD-7 and PHQ-9 – additional analyses using the IAPT-PS and WSAS 21](#_Toc65866319)

[References 27](#_Toc65866320)

# **Appendix S1: Recruitment, eligibility criteria and Consort diagram**

Between June 2017 to April 2018, recruited participants were new IAPT referrals, aged 18 ≥ 80 years, screened for psychological pathology using clinical thresholds for Patient-Health Questionnaire-9 (PHQ-9 ≥ 10) and/or Generalised Anxiety Disorder-7 (GAD-7 ≥ 8), and suitable for iCBT (i.e. willing to engage in iCBT, internet access). Exclusion criteria included: suicidal ideation/intended (PHQ-9 question 9 score > 2 and/or during clinical interview); psychotic illness; organic mental health disorder; alcohol and/or drug misuse; and currently receiving psychological treatment. The *Mini International Neuropsychiatric Interview 7.0.2* (M.I.N.I. 7.0.2) was administered by Psychological Wellbeing Practitioners (PWPs) to determine diagnosis of depression and/or anxiety disorder at baseline (Appendix S2) and 3-months follow-up. Of 430 consenting participants, 361 (84%) met all inclusion criteria and were randomised (Figure S1.1).


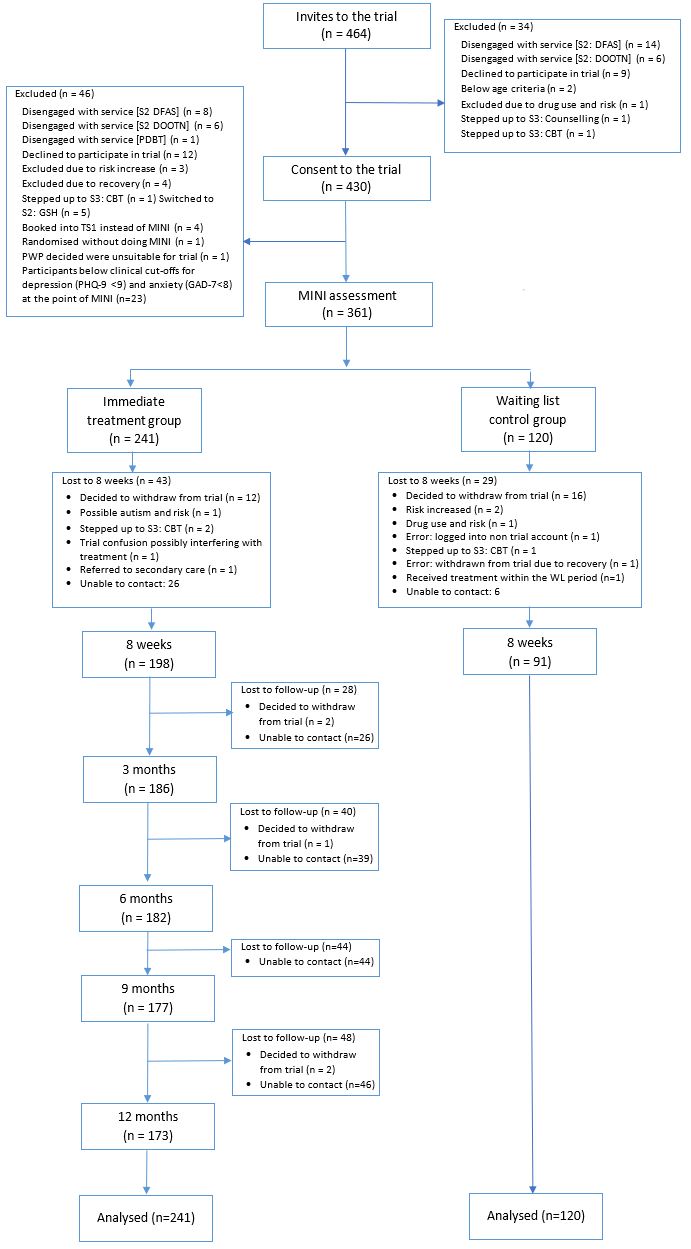


**Figure S1.1: Consort diagram**

# **Appendix S2: Baseline demographics and patient characteristics**

**Objective.** To describe the patient characteristics of those in the trial as a whole cohort and within trial-arms as reported at baseline.

**Methods.** At initial assessment with a Psychological Wellbeing Practitioners, demographic details were collected.

**Results.** Overall, 361 people were randomised (241 intervention-arm: 120 control-arm). As shown in Table S2.1, across the whole cohort the majority of participants were female (71.5%), of White/ White British and Irish ethnicity (84.2%), employed full-time (74.5%), not prescribed psychiatric medication (51.5%), and not receiving statuary sick pay (93.4%). The M.I.N.I. classified 80.3% as having major depressive (52.4%) or anxiety disorder (64.0%), with 36.0% having both. Based on Table S2.1, these characteristics are not observably different between trial-arms and were not statistically different as described and presented alongside the main trial results [1].

**Conclusion.** The trial population represents a majority of female, White/ White British and Irish, employed people, the majority of whom were diagnosed with depressive and/or anxiety disorder.

| **Demographic, value** | **Overall** | **Intervention** | **Control** |
| --- | --- | --- | --- |
| Number of people, N (%) | 361 (100%) | 241 (67%) | 120 (33%) |
| Age, mean  (median, SD,  min-max) | 33.1  (29, 12.4,  18 to 74) | 32.7  (29, 12.5,  18 to 74) | 33.9  (31, 12.1,  18 to 67) |
| Female, N (%) | 258 (71.5) | 173 (71.8) | 85 (70.8) |
| Ethnicity, N (%) |  |  |  |
| - White/ White British and Irish | 304 (84.2) | 206 (85.5) | 98 (81.7) |
| - Asian/Asian British | 23 (6.4) | 12 (5.4) | 10 (8.3) |
| - Black/African/Caribbean/Black British | 13 (3.6) | 7 (2.9) | 6 (5.0) |
| - Mixed/multiple ethnic groups | 12 (3.3) | 8 (3.3) | 4 (3.3) |
| - Other ethnic groups | 2 (0.6) | 2 (0.8) | 0 (0.0) |
| - Unknown/not stated | 7 (1.9) | 5 (2.1) | 2 (1.7) |
| Employment status, N (%) |  |  |  |
| - Employed full-time | 269 (74.5) | 184 (76.4) | 85 (70.8) |
| - Employed part-time | 0 (0.0) | 0 (0.0) | 0 (0.0) |
| - Full-time homemaker or carer | 13 (3.6) | 6 (2.5) | 7 (5.8) |
| - Full-time student | 40 (11.1) | 30 (12.5) | 10 (8.3) |
| - Long-term sick or disabled receiving benefits | 6 (1.7) | 2 (0.8) | 4 (3.3) |
| - Retired | 8 (2.2) | 6 (2.5) | 2 (1.7) |
| - Unemployed | 24 (6.7) | 12 (5.0) | 12 (10.0) |
| - Unpaid voluntary worker & not employed | 1 (0.3) | 1 (0.4) | 0 (0.0) |
| Psychiatric medication, N (%) |  |  |  |
| - Not prescribed | 186 (51.5) | 126 (52.3) | 60 (50.0) |
| - Prescribed and taking | 158 (43.8) | 99 (41.1) | 59 (49.2) |
| - Prescribed but not taking | 16 (4.4) | 15 (6.2) | 1 (0.8) |
| - Unknown | 1 (0.3) | 1 (0.4) | 0 (0.0) |
| Statutory sick pay, N (%) |  |  |  |
| - No | 337 (93.4) | 225 (93.4) | 112 (93.3) |
| - Yes | 19 (5.3) | 14 (5.8) | 5 (4.2) |
| - Not stated/unknown | 5 (1.4) | 2 (0.8) | 3 (2.5) |
| M.I.N.I. 7.02 diagnosis, N (%) | 290 (80.3) | 193 (80.1) | 97 (80.8) |
| - Major Depressive Disorder | 189 (52.4) | 125 (51.9) | 64 (53.3) |
| - Anxiety Disorder(s) | 231 (64.0) | 156 (64.7) | 75 (62.5) |
| - - Generalised Anxiety Disorder | 199 (55.1) | 134 (55.6) | 65 (54.2) |
| - - Social Anxiety Disorder | 63 (17.5) | 42 (17.4) | 21 (17.5) |
| - - Panic Disorder | 61 (16.9) | 42 (17.4) | 19 (15.8) |
| - Comorbid depressive and anxiety disorder | 130 (36.0) | 88 (36.5) | 42 (35.0) |

**Table S2.1: Baseline demographics and patient characteristics**

# **Appendix S3: Histograms of outcome measure score distributions at baseline across trial-arms**

**Objective.** To visually present the outcome measure score distributions at baseline, to inform the type of statistical tests (i.e. parametric or non-parametric tests) to be used for assessing construct validity using data from this time-point and responsiveness between time-points.

**Methods.** Histograms with a plotted normal distribution curve are used to visually assess if the scores of the outcome measures fit a normal distribution, in which case parametric tests which assume normality will be used, otherwise non-parametric tests will be used. These outcome measures scores (described within the main manuscript, Table 1, or Appendix S7) included in this assessment are: EQ-5D-5L VSE, EQ-5D-5L cross-walk, EQ-5D-5L anxiety/depression item, ReQoL-UI, ReQoL-10, PHQ-9, GAD-7, IAPT-PS items, and WSAS.

**Results.** All histograms are presented within Figures S3.1 (generic measures) and S3.2 (condition-specific and functional impairment). The EQ-5D-5L (VSE and cross-walk) and ReQoL-UI measure scores don’t fit a normal distribution, with bi-, or tri-, modal peaks. The EQ-5D-5L anxiety/depression item and ReQoL-10 better fits a normal distribution than the associated within measure preference-based scores. The PHQ-9, GAD-7, and WSAS also better fit a normal distribution, with the IAPT-PS item of social anxiety having a much different distribution to panic disorder or social phobia items.

**Conclusion**. Although the condition-specific and ReQoL-10 summary scores reasonably fitted a normal distribution, this is not generally the case for the other preference-based or item scores. As such and for cross-comparison, non-parametric tests will be used for psychometric assessment.


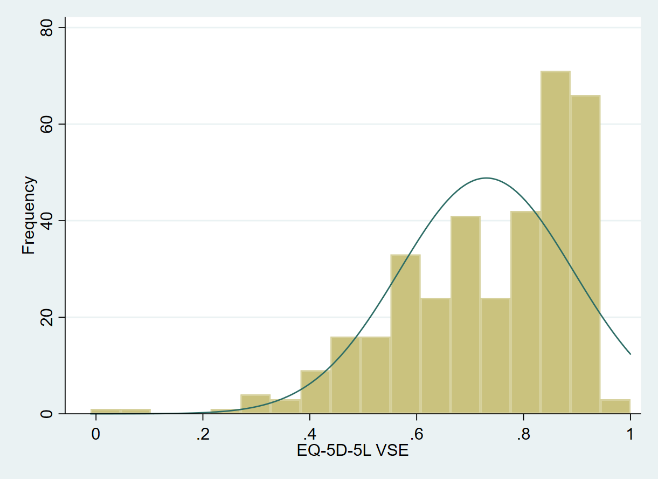

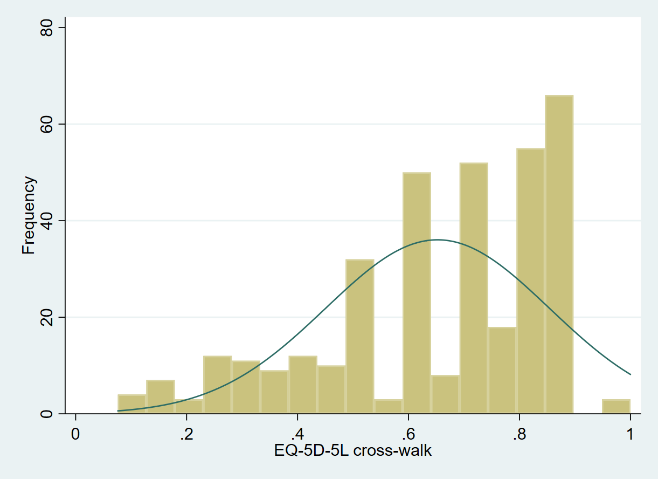


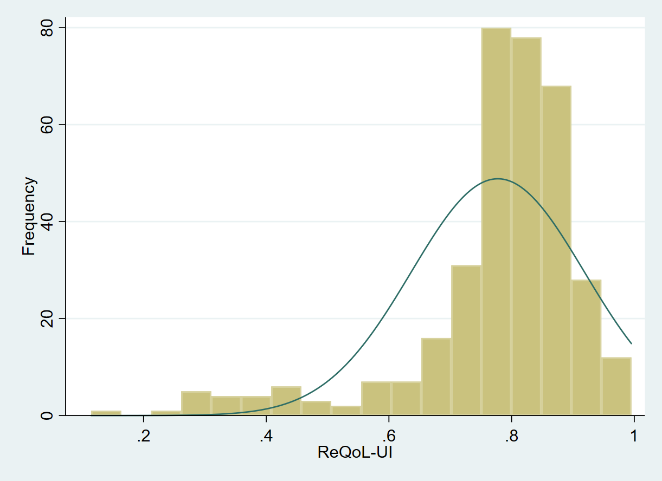
 **
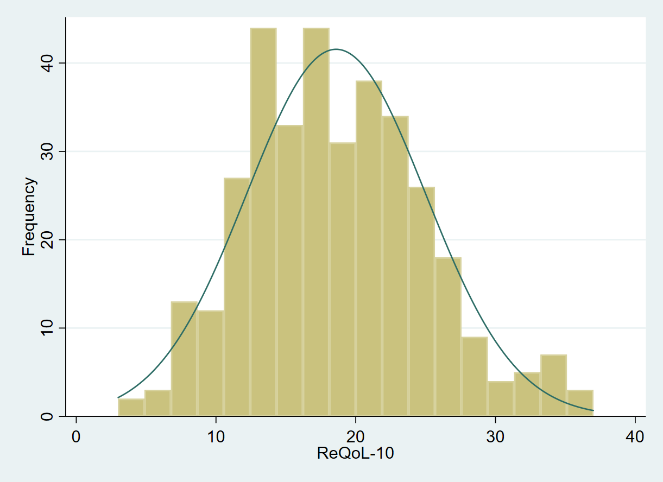
**
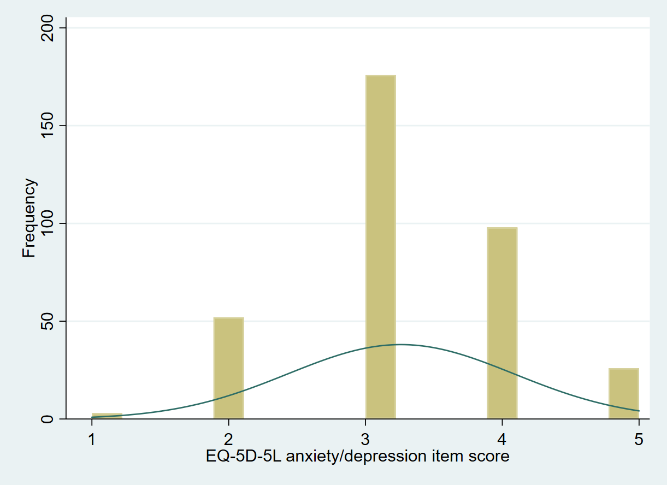


**Figure S3.1: EQ-5D-5L VSE and cross-walk, anxiety/depression item, ReQoL-UI, and ReQoL-10 score distributions**


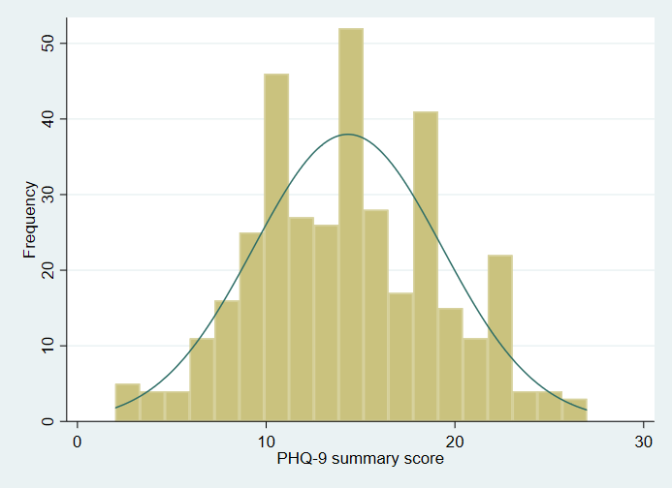

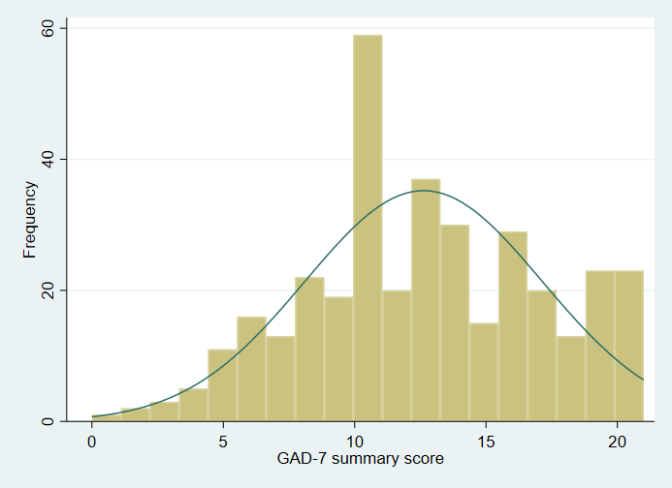


**
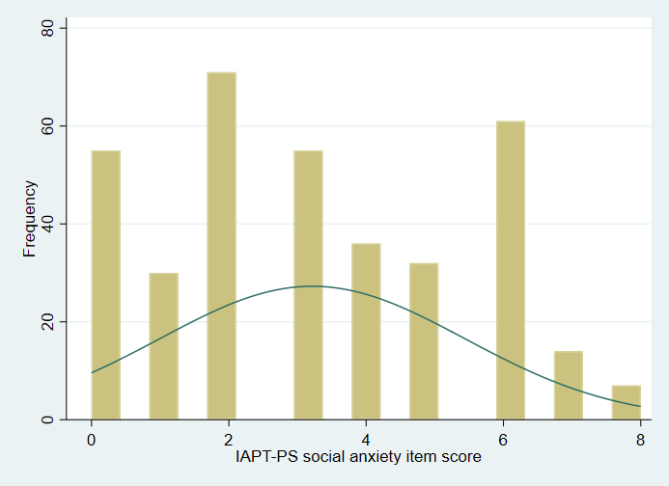
** **
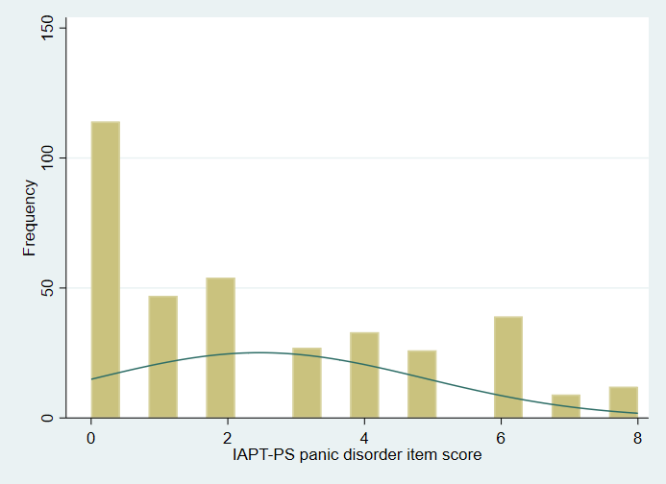
**

**
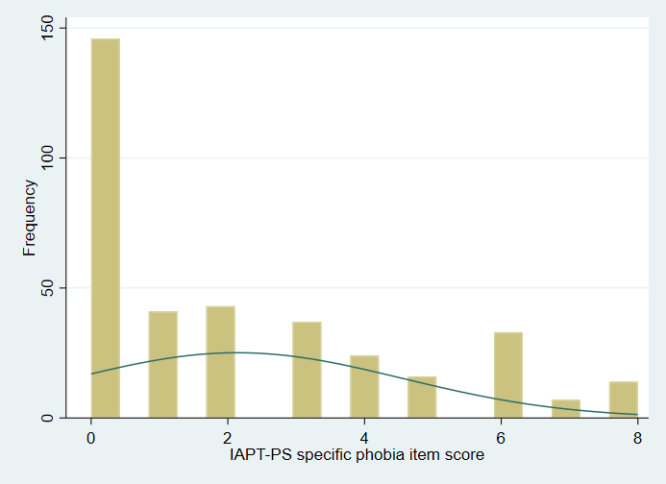
** **
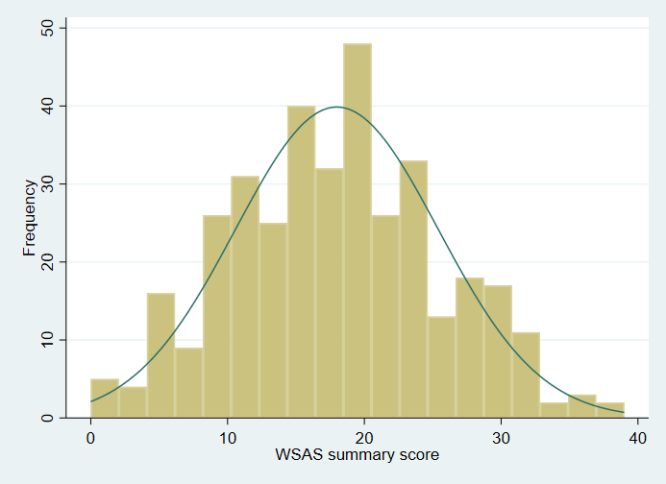
**

**Figure S3.2: PHQ-9, GHQ-7, IAPT-PS items (social anxiety, panic disorder, specific phobia), and WSAS score distributions**

# **Appendix S4: Generic measure scores including ceiling and floor effects by trial-arm and time-point**

**Objective.** To describe ceiling and floor effects for the generic measures to accompany the assessment of responsiveness as described in the main manuscript.

**Methods.** Ceiling and floor effects (as described in the main manuscript) are assessed by trial-arm (intervention and waiting-list control) at all available data collection time-points (baseline, 8-week; intervention-arm, also 3-, 6-, 9-, and 12-month) for the EQ-5D-5L (VSE and cross-walk), EQ-5D-5L anxiety/depression item, ReQoL-UI, and ReQoL-10 scores. Additional score statistics (number of responders, mean, standard deviation, median, observed worst and best score) are also provided to aid the reader without having to refer back to similar summary statistics presented in Table 3 of the main manuscript.

**Results.** Ceiling and floor effects are presented in Table S4.1 (intervention-arm) and Table S4.2 (control-arm). The results generally suggest that a lower proportion of people reported the best possible state on the ReQoL-UI value set score than on the EQ-5D-5L VSE and cross-walk scores at all time-points in both the intervention and control trial-arms, suggesting the ReQoL-UI value set score generally doesn’t particularly suffer from ceiling effects, which were more present for the EQ-5D-5L VSE and cross-walk scores at follow-up time-points (in particular, at 9-month in the intervention-arm). Ceiling effects were also less present for the ReQoL-10 than ReQoL-UI. Ceiling effects were more present in the EQ-5D-5L anxiety/depression item; although, this represents no problem with anxiety/depression which is an intended and desirable outcome from the intervention assessed in the RCT from which the data was provided.

**Conclusion**. The ReQoL-UI (and ReQoL-10) has a low presence of ceiling effects, which is lower than for the EQ-5D-5L VSE and cross-walk scores. This suggests the ReQoL-UI (and ReQoL-10) has a better ability to be responsive to an improvement in preference-based health status than the EQ-5D-5L VSE and cross-walk.

**Table S4.1: Outcome measure score, floor and ceiling effects within the intervention-arm across time-points**

| **Time-point** | **Time-point** | **N (%)** | **Mean** | **Median** | **SD** | **P. floor/**  **worst score** | **P. ceiling/**  **best score** | **O.**  **worst score** | **O.**  **best score** | **N floor/**  **worst score (%)** | **N ceiling/**  **best score (%)** |
| --- | --- | --- | --- | --- | --- | --- | --- | --- | --- | --- | --- |
| EQ-5D-5L VSE | Baseline | 238 (99) | 0.735 | 0.783 | 0.152 | -0.285 | 1 | 0.089 | 1 | 0 (0.0) | 2 (0.8) |
|  | 8-week | 198 (82) | 0.794 | 0.833 | 0.147 | -0.285 | 1 | 0.270 | 1 | 0 (0.0) | 8 (4.0) |
|  | 3-month | 186 (77) | 0.816 | 0.859 | 0.149 | -0.285 | 1 | 0.110 | 1 | 0 (0.0) | 15 (8.1) |
|  | 6-month | 182 (76) | 0.830 | 0.892 | 0.171 | -0.285 | 1 | 0.089 | 1 | 0 (0.0) | 28 (15.4) |
|  | 9-month | 176 (73) | 0.837 | 0.892 | 0.170 | -0.285 | 1 | 0.090 | 1 | 0 (0.0) | 33 (18.8) |
|  | 12-month | 172 (71) | 0.814 | 0.859 | 0.172 | -0.285 | 1 | 0.062 | 1 | 0 (0.0) | 21 (12.2) |
| EQ-5D-5L cross-walk | Baseline | 238 (99) | 0.656 | 0.718 | 0.193 | -0.594 | 1 | 0.119 | 1 | 0 (0.0) | 2 (0.8) |
|  | 8-week | 198 (82) | 0.723 | 0.768 | 0.182 | -0.594 | 1 | 0.028 | 1 | 0 (0.0) | 8 (4.0) |
|  | 3-month | 186 (77) | 0.753 | 0.812 | 0.180 | -0.594 | 1 | -0.086 | 1 | 0 (0.0) | 15 (8.1) |
|  | 6-month | 182 (76) | 0.767 | 0.837 | 0.212 | -0.594 | 1 | -0.058 | 1 | 0 (0.0) | 28 (15.4) |
|  | 9-month | 176 (73) | 0.779 | 0.837 | 0.204 | -0.594 | 1 | -0.092 | 1 | 0 (0.0) | 33 (18.8) |
|  | 12-month | 172 (71) | 0.751 | 0.819 | 0.201 | -0.594 | 1 | -0.116 | 1 | 0 (0.0) | 21 (12.2) |
| ReQoL-UI | Baseline | 237 (98) | 0.788 | 0.806 | 0.123 | -0.195 | 1 | 0.242 | 0.979 | 0 (0.0) | 0 (0.0) |
|  | 8-week | 198 (82) | 0.810 | 0.841 | 0.140 | -0.195 | 1 | 0.134 | 0.996 | 0 (0.0) | 0 (0.0) |
|  | 3-month | 186 (77) | 0.836 | 0.873 | 0.151 | -0.195 | 1 | 0.202 | 1 | 0 (0.0) | 3 (1.6) |
|  | 6-month | 182 (76) | 0.840 | 0.874 | 0.144 | -0.195 | 1 | 0.254 | 1 | 0 (0.0) | 4 (2.2) |
|  | 9-month | 176 (73) | 0.863 | 0.895 | 0.124 | -0.195 | 1 | 0.274 | 1 | 0 (0.0) | 4 (2.3) |
|  | 12-month | 172 (71) | 0.850 | 0.874 | 0.135 | -0.195 | 1 | 0.231 | 1 | 0 (0.0) | 7 (4.1) |
| ReQoL-10 | Baseline | 237 (98) | 18.519 | 18 | 6.237 | 0 | 40 | 3 | 35 | 0 (0.0) | 0 (0.0) |
|  | 8-week | 198 (82) | 20.995 | 21 | 6.846 | 0 | 40 | 6 | 39 | 0 (0.0) | 0 (0.0) |
|  | 3-month | 186 (77) | 24.102 | 24 | 7.359 | 0 | 40 | 0 | 40 | 1 (0.5) | 2 (1.1) |
|  | 6-month | 182 (76) | 24.593 | 25 | 7.899 | 0 | 40 | 4 | 40 | 0 (0.0) | 3 (1.6) |
|  | 9-month | 176 (73) | 25.494 | 26 | 8.200 | 0 | 40 | 8 | 40 | 0 (0.0) | 6 (3.4) |
|  | 12-month | 172 (71) | 24.541 | 25 | 8.314 | 0 | 40 | 6 | 40 | 0 (0.0) | 4 (2.3) |
| EQ-5D-5L depression/anxiety | Baseline | 238 (99) | 3.273 | 3 | 0.830 | 5 | 1 | 5 | 1 | 18 (7.6) | 2 (0.8) |
|  | 8-week | 198 (82) | 2.641 | 3 | 0.944 | 5 | 1 | 5 | 1 | 8 (4.0) | 18 (9.1) |
|  | 3-month | 186 (77) | 2.441 | 2 | 0.869 | 5 | 1 | 5 | 1 | 3 (1.6) | 22 (11.8) |
|  | 6-month | 182 (76) | 2.297 | 2 | 0.963 | 5 | 1 | 5 | 1 | 5 (2.7) | 37 (20.3) |
|  | 9-month | 176 (73) | 2.227 | 2 | 0.988 | 5 | 1 | 5 | 1 | 3 (1.7) | 45 (25.6) |
|  | 12-month | 172 (71) | 2.308 | 2 | 0.981 | 5 | 1 | 5 | 1 | 3 (1.7) | 37 (21.5) |

**Acronyms.** EQ-5D-5L, EQ-5D five-level version; N, number of responder; O., observed; P., possible; ReQoL-10, Recovering Quality of Life – 10 item; ReQoL-UI, Recovering Quality of Life – Utility Index; SD, standard deviation.

**Footnote.** The table shows the possible (P.) floor/worst and ceiling/best scores as well as the observed (O.) worst and best scores achieved by the respondents; these are shown rather than possible and observed minimum and maximum scores due to the fact for the tariff scores a higher score is a better state, whereas for the summary scores the opposite is true (i.e. a higher score is a worst state)

**Table S4.2: Outcome measure score, floor and ceiling effects within the control-arm across time-points**

| **Time-point** | **Time-point** | **N (%)** | **Mean** | **Median** | **SD** | **P. floor/**  **worst score** | **P. ceiling/**  **best score** | **O.**  **worst score** | **O.**  **best score** | **N floor/**  **worst score (%)** | **N ceiling/**  **best score (%)** |
| --- | --- | --- | --- | --- | --- | --- | --- | --- | --- | --- | --- |
| EQ-5D-5L VSE | Baseline | 117 (98) | 0.722 | 0.783 | 0.182 | -0.285 | 1 | -0.010 | 1 | 0 (0.0) | 1 (0.9) |
|  | 8-week | 91 (76) | 0.756 | 0.833 | 0.181 | -0.285 | 1 | 0.185 | 1 | 0 (0.0) | 3 (3.3) |
| EQ-5D-5L cross-walk | Baseline | 117 (98) | 0.645 | 0.721 | 0.218 | -0.594 | 1 | 0.076 | 1 | 0 (0.0) | 1 (0.9) |
|  | 8-week | 91 (76) | 0.676 | 0.750 | 0.231 | -0.594 | 1 | -0.047 | 1 | 0 (0.0) | 3 (3.3) |
| ReQoL-UI | Baseline | 116 (97) | 0.757 | 0.808 | 0.171 | -0.195 | 1 | 0.115 | 0.995 | 0 (0.0) | 0 (0.0) |
|  | 8-week | 91 (76) | 0.793 | 0.832 | 0.163 | -0.195 | 1 | 0.124 | 0.976 | 0 (0.0) | 0 (0.0) |
| ReQoL-10 | Baseline | 116 (97) | 18.759 | 19 | 6.747 | 0 | 40 | 3 | 37 | 0 (0.0) | 0 (0.0) |
|  | 8-week | 91 (76) | 20.253 | 21 | 6.402 | 0 | 40 | 6 | 40 | 0 (0.0) | 1 (1.1) |
| EQ-5D-5L depression/anxiety | Baseline | 117 (98) | 3.231 | 3 | 0.824 | 5 | 1 | 5 | 1 | 8 (6.8) | 1 (0.9) |
|  | 8-week | 91 (76) | 2.912 | 3 | 1.007 | 5 | 1 | 5 | 1 | 8 (8.8) | 5 (5.5) |

**Acronyms.** EQ-5D-5L, EQ-5D five-level version; N, number of responder; O., observed; P., possible; ReQoL-10, Recovering Quality of Life – 10 item; ReQoL-UI, Recovering Quality of Life – Utility Index; SD, standard deviation.

**Footnote.** The table shows the possible (P.) floor/worst and ceiling/best scores as well as the observed (O.) worst and best scores achieved by the respondents; these are shown rather than possible and observed minimum and maximum scores due to the fact for the tariff scores a higher score is a better state, whereas for the summary scores the opposite is true (i.e. a higher score is a worst state)

# **Appendix S5: Assessing Convergent validity using locally weighted scatterplot smoothing (LOWESS) techniques**

**Objective.** To assess the convergent validity of the EQ-5D-5L VSE and cross-walk, EQ-5D-5L anxiety/depression item, ReQoL-UI, and ReQoL-10 against condition-specific (PHQ-9, GAD-7, and IAPT-PS items) and functional impairment (WSAS) measures.

**Methods.** Convergent validity assesses the relationship strength between measures which can be assessed visually using locally weighted scatterplot smoothing (LOWESS) techniques, which also complements the correlation analysis described in the main manuscript. LOWESS is a form of non-parametric regression which plots a line of central tendency between two variables on a scatterplot, thereby visualising their general relationship across the possible score ranges without making assumptions about the actual relationship [2]. These LOWESS lines are based on outcome measure data collected at baseline across the whole cohort.

**Results.** The LOWESS figures are presented in Figures S5.1 (EQ-5D-5L VSE), S5.2 (EQ-5D-5L cross-walk), S5.3 (EQ-5D-5L anxiety/depression item), S5.4 (ReQoL-UI), and S5.5 (ReQoL-10). In general, the LOWESS figures complements the relationship evidence from assessing correlation strength as described in the main manuscript and Table 4, particularly in terms of direction of the relationship (e.g. downward or upward trends) while allowing a visual assessment of the relationship at different intervals of the measures scores. For example, across all the EQ-5D-5L VSE (Figure S5.1) and cross-walk (Figure S5.2), and ReQoL-UI (Figure S5.4) the downward trend indicates that as a person’s condition-specific or functional impairment severity worsens (a worse state represented by a higher score on these measures) as their preference-based health declines (represented by a lower preference-based score). When assessing the EQ-5D-5L anxiety/depression item against GAD-7 and PHQ-9 (Figure S5.3), a notable upward trend is observed with the GAD-7 (i.e. as anxiety severity worsens as represented by the GAD-7, anxiety/depression severity worsens on the EQ-5D-5L anxiety/depression item across the majority of the scores ranges) but this upward trend is only observed at the higher scores on the PHQ-9 (i.e. as depression severity worsens as represented by the PHQ-9, the relationship is only notable with a change in EQ-5D-5L anxiety/depression item at the more severe end of the PHQ-9, e.g. PHQ-9≥15). In relation to this observed relationship between PHQ-9 and EQ-5D-5L anxiety/depression item, this could explain the worse correlation strength with these measures than the EQ-5D-5L anxiety/depression item with GAD-7, as well as the higher effect sizes for EQ-5D-5L anxiety/depression item with PHQ-9 “Severe” (i.e. ≥20) cut-off category than the other PHQ-9 severity cut-off categories (see Table 5). We also see a much more pronounced downward trend with the ReQoL-10 and the PHQ-9 than with the GAD-7 across the whole scores scale, which reinforces the correlation strengths suggested in Table 4.

**Conclusion.** These LOWESS lines complement and support the correlation strengths described in the main manuscript, while providing a useful visual presentation of the relationship across the scores ranges of the two measures being compared.


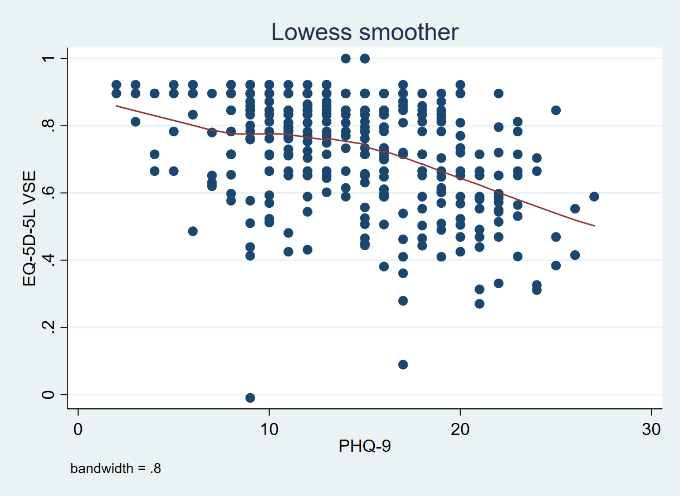

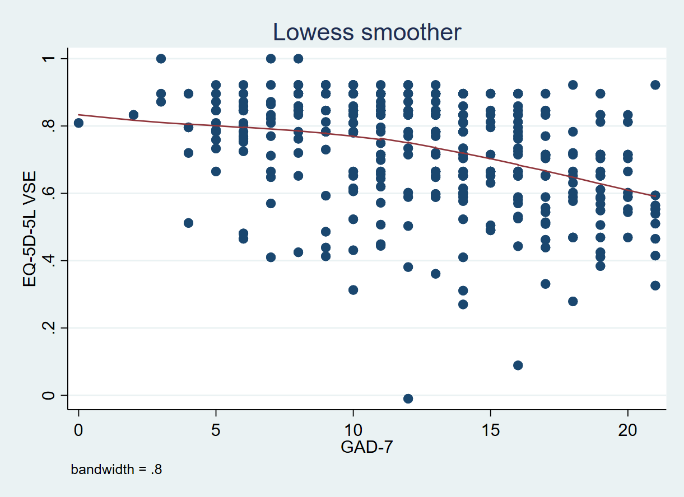


**
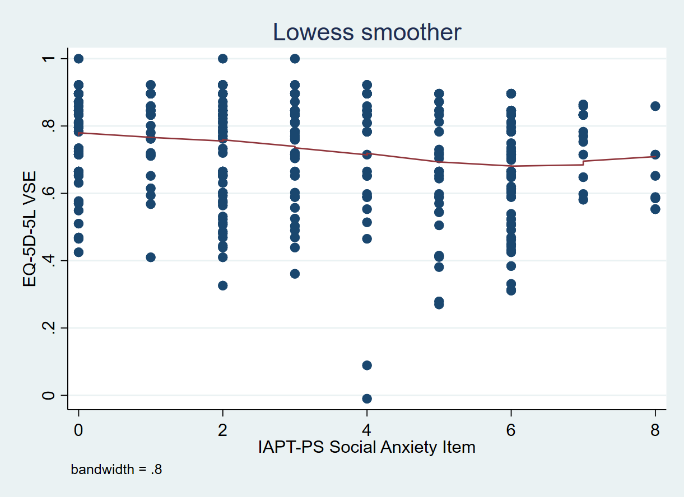
**  **
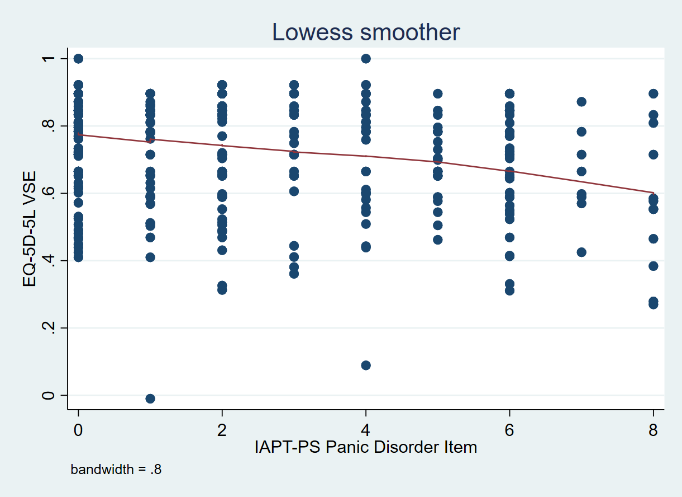
**

**
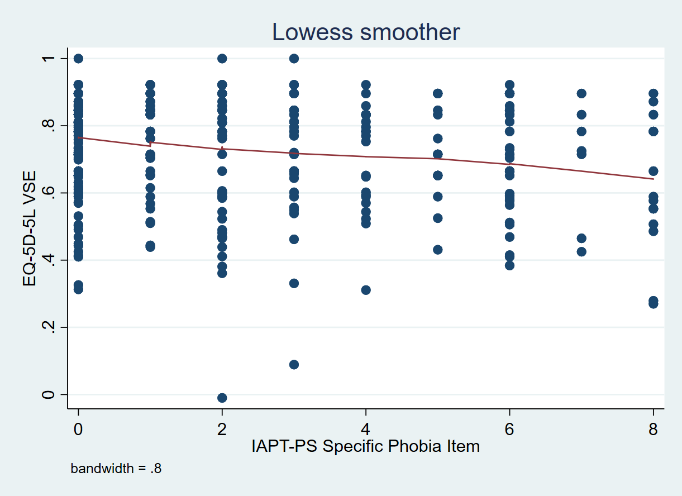
**
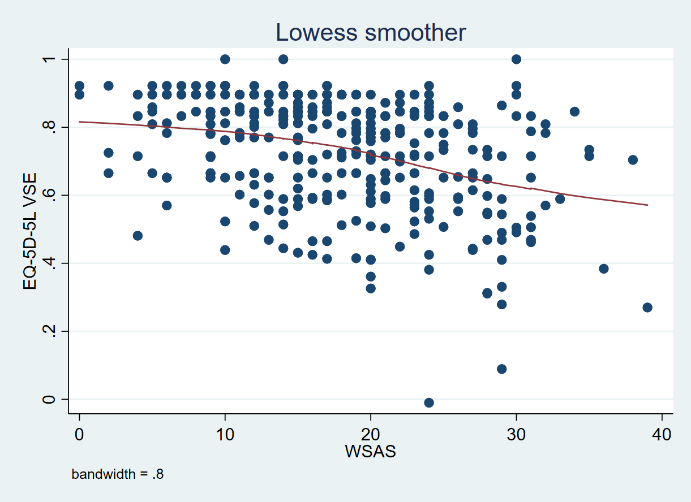


**Figure S5.1: LOWESS of EQ-5D-5L VSE against condition-specific and functional impairment measures**


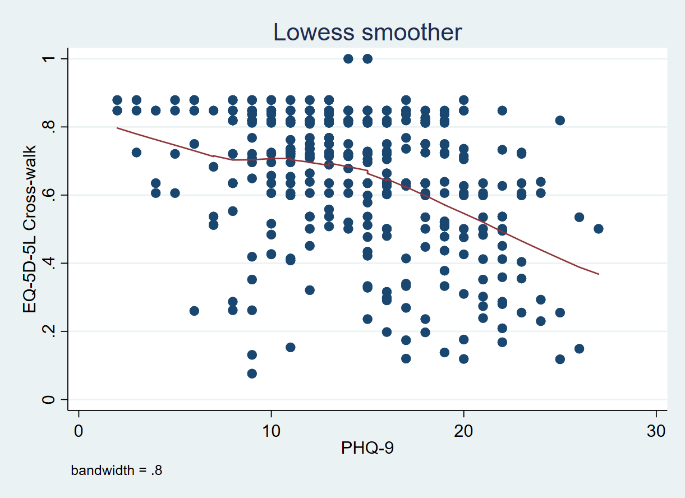

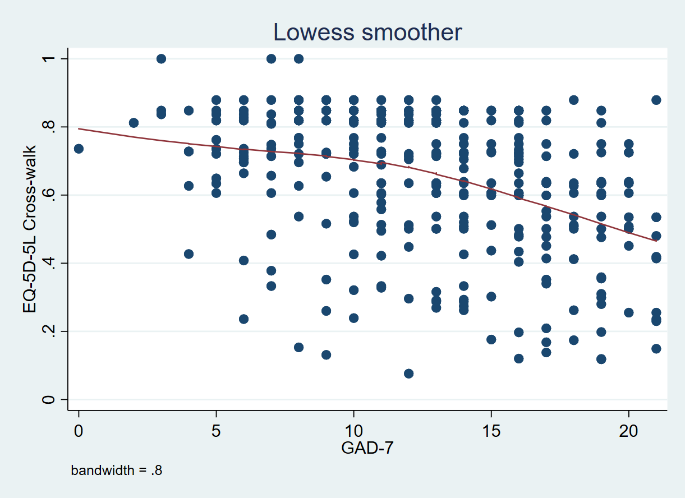


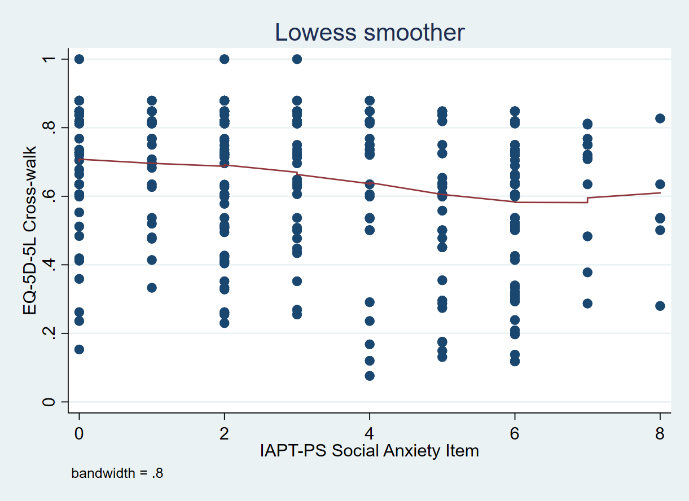

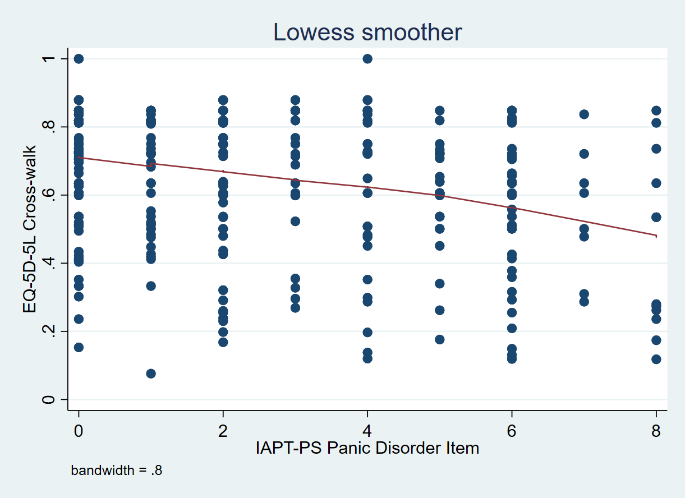


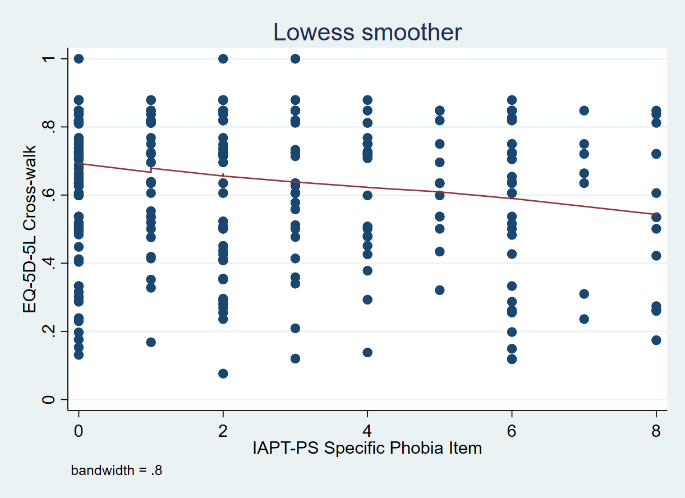

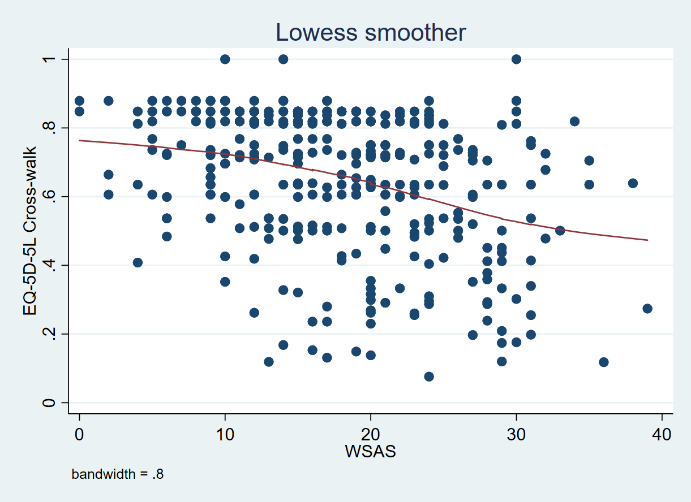


**Figure S5.2: LOWESS of EQ-5D-5L cross-walk against condition-specific and functional impairment measures**


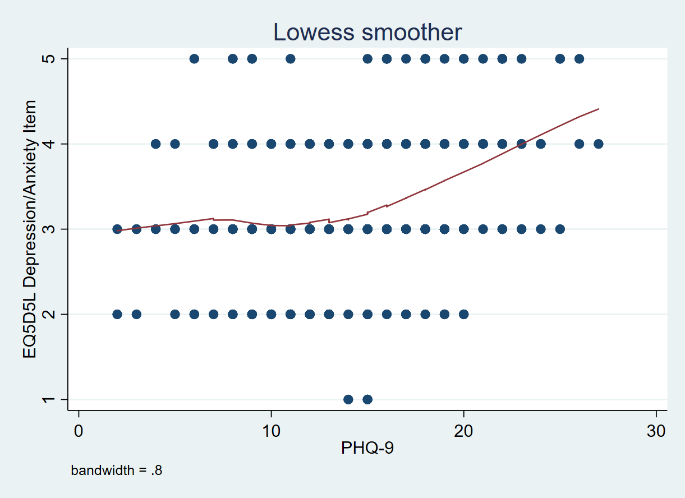

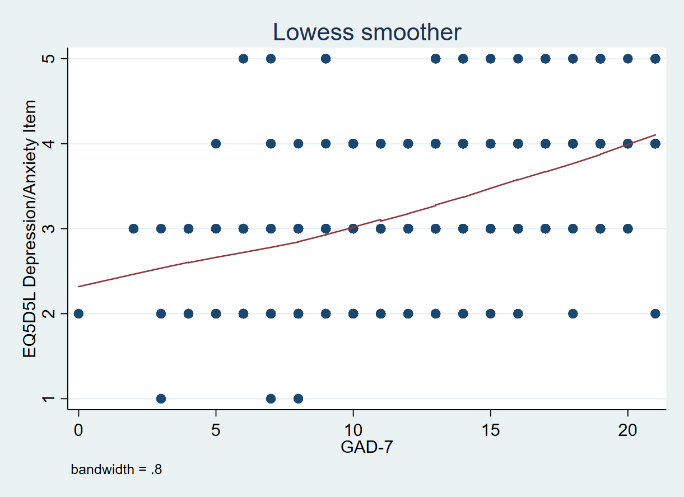


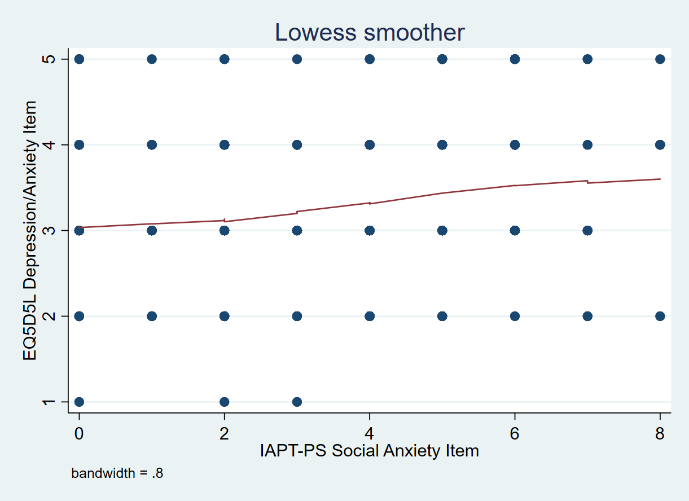

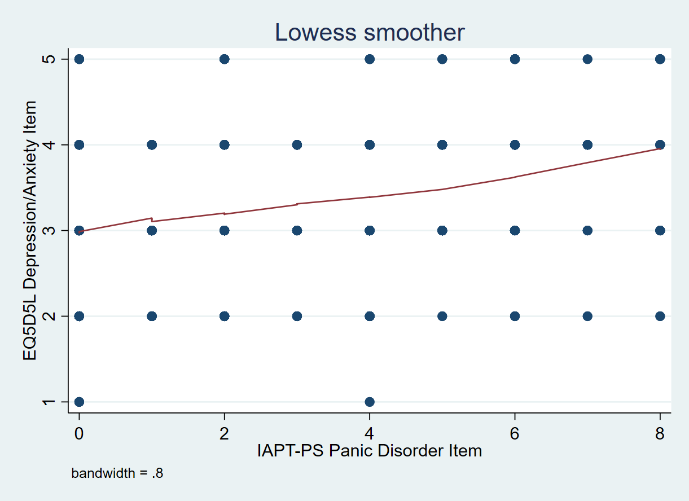


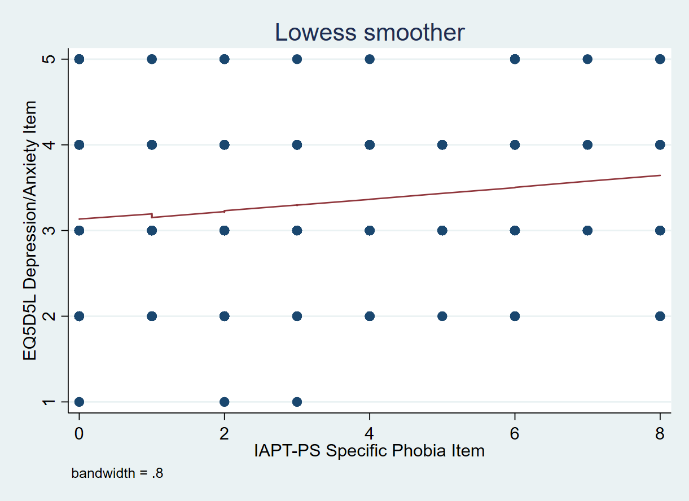

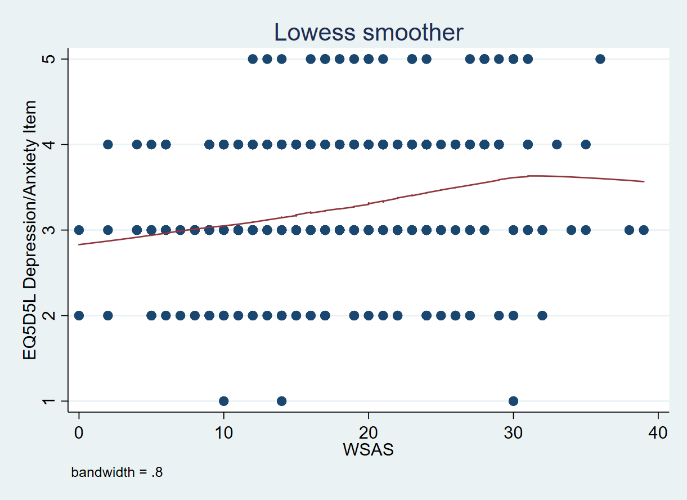


**Figure S5.3: LOWESS of EQ-5D-5L anxiety/depression item against condition-specific and functional impairment measures**


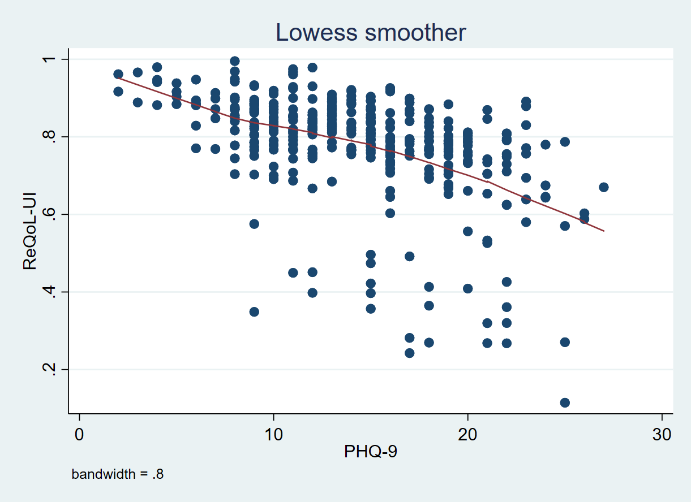

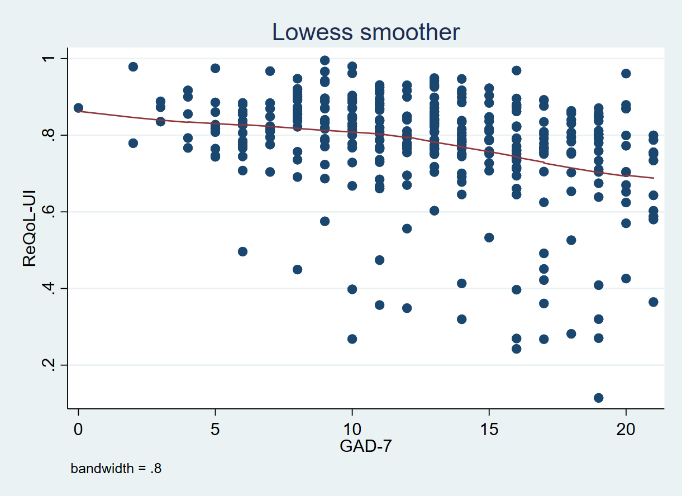


**
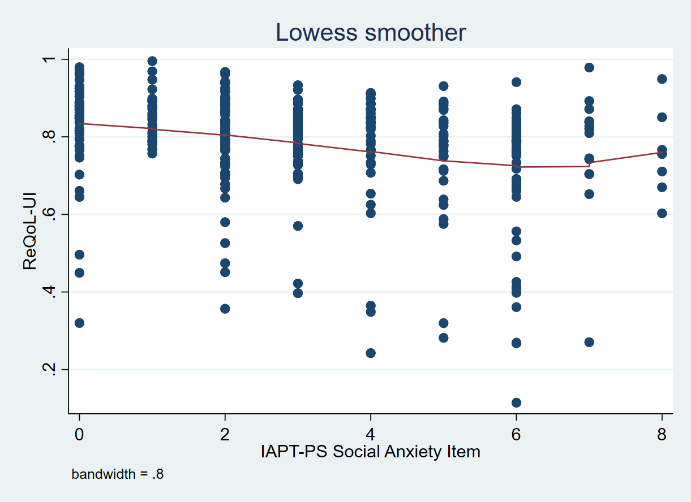
**  **
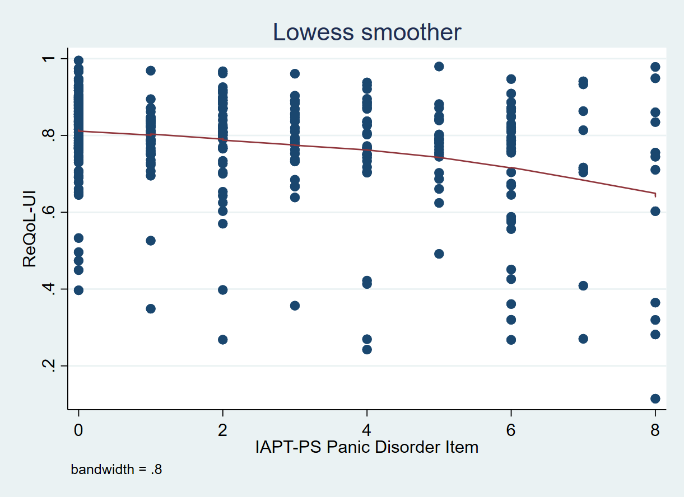
**

**
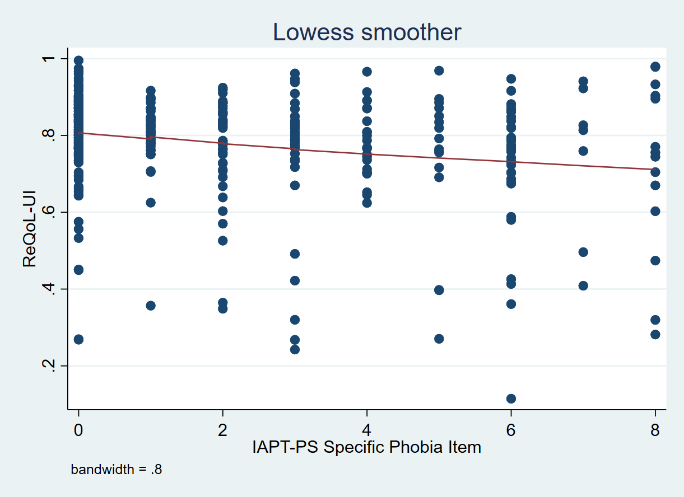
**  **
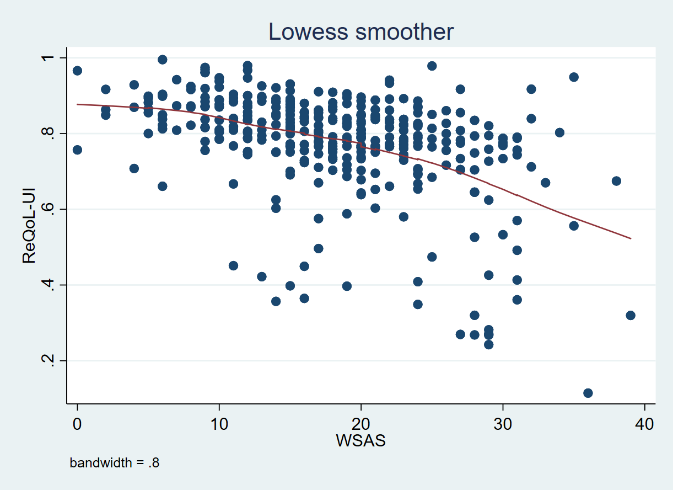
**

**Figure S5.4: LOWESS of ReQoL-UI against condition-specific and functional impairment measures**


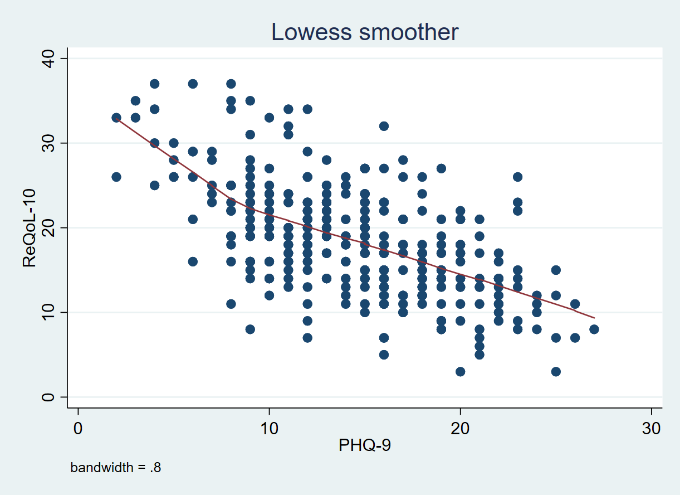

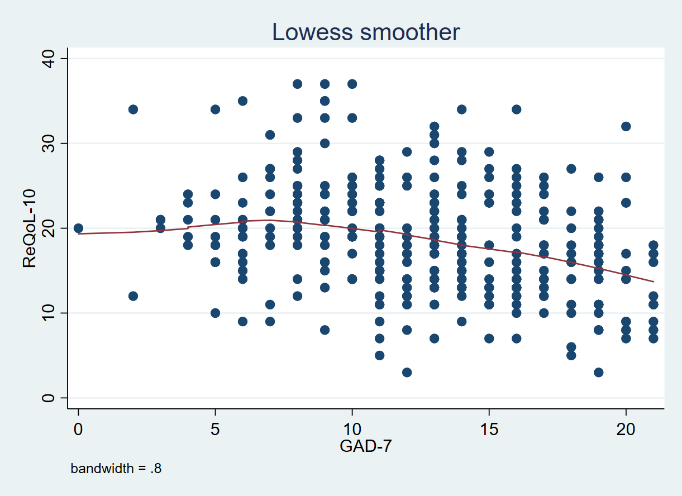


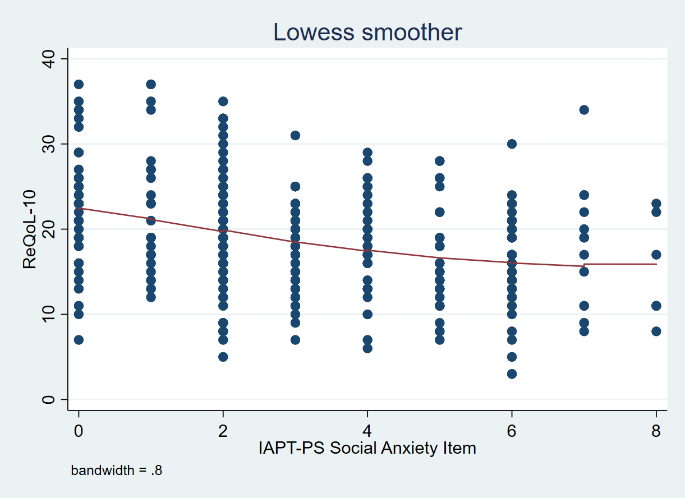

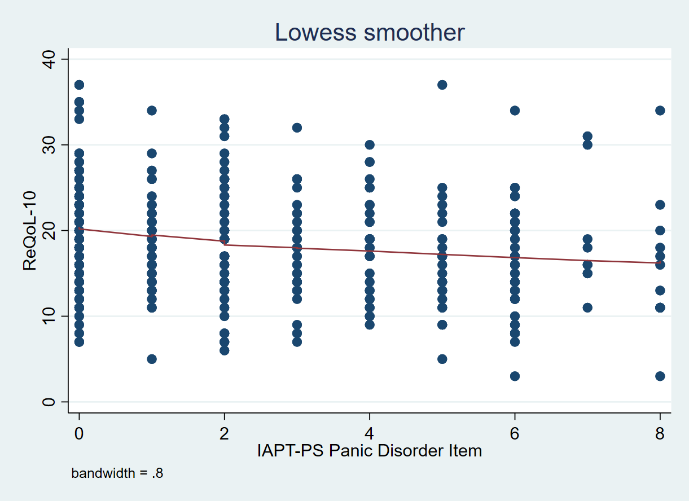


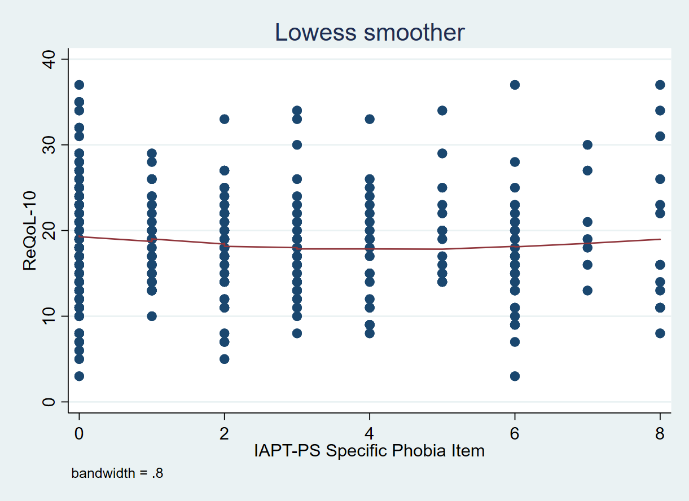

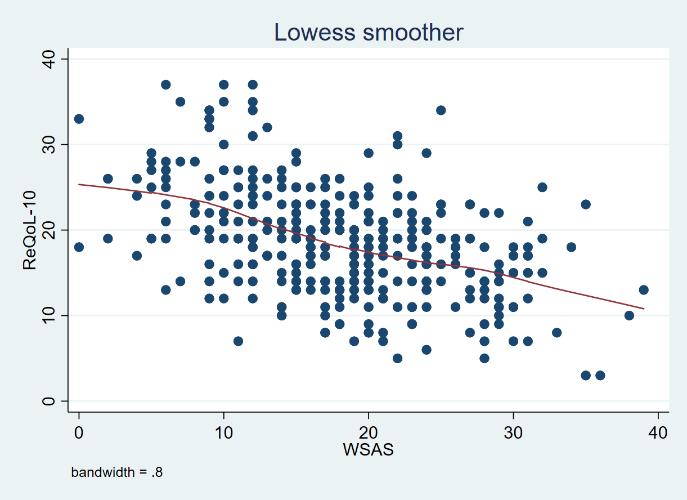


**Figure S5.5: LOWESS of ReQoL-10 against condition-specific and functional impairment measures**

# **Appendix S6: Assessing Convergent validity at the item-level – a complementary analysis**

**Background.** Overall outcome measure scores are, in part, a composite of the individual items included within these measures. Although the overall scores are designed to represent the severity of an overall construct (i.e. preference-based health, anxiety and/or depression severity), exploring the relationships between the items (and summary or preference-based scores) and the domains they represent can further help inform what aspects of the measures may be influencing the overall relationship between measures.

**Objective.** To explore the convergent validity between the EQ-5D-5L, ReQoL-UI/-10, PHQ-9, and GAD-7 at the item-level, which will inform a discussion about how individual items may be influencing the convergent validity relationship described in the main manuscript at the preference-based and summary score level.

**Methods.** Correlation analysis, here, indicates the degree to which the instruments are measuring related factors at mainly the item level (although total summary and tariff scores are included for analysis). Absolute Correlation Strength (ACS) is: weak, <0.3; moderate, 0.3 <0.5; and strong, >0.5 [3]; for the purpose of discussion here, we will focus mainly on those correlations which are moderate or strong in strength. Spearmans rank correlation coefficient as a non-parametric test is used. Due to the large number of items within measures and, thus, use of multiple hypotheses tests, statistical significance (SS) is defined here as p < 0.01; we have chosen a lower SS threshold as to refine the focus and discussion of SS results.

**Results.** Descriptive statistics of baseline item (summary and preference-based) scores are provided in Table S6.1 (Table 1 provides an overview of how to interpret these scores). The correlation matrix between the EQ-5D-5L and ReQoL-10 items (including preference-based and summary scores) with the PHQ-9 is shown in Table S6.2 and with GAD-7 in Table S6.3. The lack of moderate or strong, and SS, correlations are more notable with the GAD-7 than PHQ-9.

With the GAD-7, it is apparent that the EQ-5D-5L’s moderate convergent validity is mainly attributable to the anxiety/depression item (mainly moderate and SS ACS with all GAD-7 items apart from ‘afraid’ which is weak and SS) as all other EQ-5D-5L items have mainly non-SS and/or weak ACS with all GAD-7 items and the summary score. For the ReQoL-10/-UI items, all ACS are weak with mixed results in terms of SS at the GAD-7 item-level, although all results are SS at the GAD-7 summary score level. The GAD-7 item of ‘trouble relaxing’ has the most number of SS ACS with the ReQoL-10 items (all SS apart from with ‘trust others’ and ‘life not worth living’), with a moderate strength and SS ACS with the ReQoL-10 and ReQoL-UI score (noting that ‘trust others’ is not part of the ReQoL-UI items).

In comparison with the PHQ-9, it becomes apparent why the ReQoL-10/-UI has a stronger convergent validity with the PHQ-9 than GAD-7 as at the item-level, the number of SS ACS become more notable and although most are of weak strength, there are some of moderate and strong strength. For example, six of the ReQoL-10 (three of the ReQoL-UI) items have a moderate or strong (one strong with ‘starting daily tasks’ ReQoL-10 item) and SS ACS with the PHQ-9 item of ‘interest/ pleasure in doing things’, with the PHQ-9 item having a moderate and SS correlation with both the ReQoL-10 summary score and ReQoL-UI item score (albeit the ACS strength is weaker for the ReQoL-UI than ReQoL-10). The only other strong ACS is between the ReQoL-10/-UI item of ‘life not worth living’ and PHQ-9 item of ‘suicidal / self-harm thoughts’. For the EQ-5D-5L, although the ACS are all SS between all PHQ-9 items and EQ-5D-5L items of ‘usual activities’ and ‘anxiety/depression’, these are mostly weak ACS (only exception is between ‘usual activities’ and ‘interest/pleasure in doing things’, which is of moderate strength), with a moderate ACS with the PHQ-9 summary score; the EQ-5D-5L items of ‘mobility’ and ‘pain/discomfort’ all have non-SS ACS with the PHQ-9 items and summary score.

**Conclusion.** In the main manuscript we concluded that the ReQoL-UI/-10 had better construct validity with depression (PHQ-9) and EQ-5D-5L (VSE and cross-walk) better with anxiety (GAD-7) severity. At the item-level, it is apparent that the EQ-5D-5L’s convergent validity with GAD-7 comes mainly from the anxiety/depression item, which has SS and mainly moderate ACS at the GAD-7 item level and strong ACS at the GAD-7 summary score level. The ReQoL-10/-UI items have much more SS and moderate/strong strength ACS with the PHQ-9 items/summary scores than the EQ-5D-5L, and much more than with the GAD-7 items/summary scores. At the item-level, it is apparent which items are driving the overall convergent validity relationship as part for the measures descriptive system; however for the preference-based measures, the effect the value sets and cross-walk scores has on this relationship is not fully assessed within this analysis.

**Table S6.1: Baseline outcome measure item, summary and value set score statistics**

| **Measure** | **Items/score** | **N (%)** | **Mean** | **Median** | **SD** | **Min** | **Max** |
| --- | --- | --- | --- | --- | --- | --- | --- |
| PHQ-9 | - Interest/ pleasure in doing things | 361 (100) | 1.67 | 2 | 0.85 | 0 | 3 |
|  | - Down/ depressed/ hopeless | 361 (100) | 1.77 | 2 | 0.86 | 0 | 3 |
|  | - Sleep | 361 (100) | 2.11 | 2 | 0.96 | 0 | 3 |
|  | - Tired/ energy | 361 (100) | 2.22 | 2 | 0.83 | 0 | 3 |
|  | - Poor appetite/overeating | 361 (100) | 1.70 | 2 | 1.08 | 0 | 3 |
|  | - Feeling bad/ failure | 361 (100) | 1.81 | 2 | 0.96 | 0 | 3 |
|  | - Concentration | 361 (100) | 1.65 | 2 | 0.97 | 0 | 3 |
|  | - Moving or speaking slowly OR fidgety/restless | 361 (100) | 0.93 | 1 | 0.95 | 0 | 3 |
|  | - Suicidal/ self-harm thoughts | 361 (100) | 0.47 | 0 | 0.75 | 0 | 3 |
|  | Summary score | 361 (100) | 14.33 | 14 | 4.99 | 2 | 27 |
| GAD-7 | - Nervous/ anxious | 361 (100) | 2.12 | 2 | 0.86 | 0 | 3 |
|  | - Stop/ control worrying | 361 (100) | 2.08 | 2 | 0.88 | 0 | 3 |
|  | - Worrying about things | 361 (100) | 2.14 | 2 | 0.85 | 0 | 3 |
|  | - Trouble relaxing | 361 (100) | 1.90 | 2 | 0.86 | 0 | 3 |
|  | - Restless | 361 (100) | 1.10 | 1 | 0.98 | 0 | 3 |
|  | - Annoyed/irritable | 361 (100) | 1.79 | 2 | 0.94 | 0 | 3 |
|  | - Afraid | 361 (100) | 1.51 | 1 | 1.08 | 0 | 3 |
|  | Summary score | 361 (100) | 12.62 | 13 | 4.52 | 0 | 21 |
| EQ-5D-5L | - Mobility | 355 (98) | 1.19 | 1 | 0.57 | 1 | 5 |
|  | - Self-care | 355 (98) | 1.19 | 1 | 0.50 | 1 | 4 |
|  | - Usual activities | 355 (98) | 2.02 | 2 | 0.98 | 1 | 5 |
|  | - Pain/discomfort | 355 (98) | 1.71 | 1 | 0.91 | 1 | 5 |
|  | - Anxiety/depression | 355 (98) | 3.26 | 3 | 0.83 | 1 | 5 |
|  | VSE | 355 (98) | 0.730 | 0.783 | 0.163 | -0.010 | 1 |
|  | Cross-walk | 355 (98) | 0.652 | 0.721 | 0.202 | 0.076 | 1 |
| ReQoL-10^1^ | - Starting daily tasks | 353 (98) | 1.61 | 2 | 1.01 | 0 | 4 |
|  | - Trust others | 353 (98) | 2.05 | 2 | 1.07 | 0 | 4 |
|  | *- Unable to cope* | 353 (98) | 1.74 | 2 | 0.96 | 0 | 4 |
|  | - Doing things | 353 (98) | 1.99 | 2 | 0.95 | 0 | 4 |
|  | *- Happy* | 353 (98) | 1.70 | 2 | 0.85 | 0 | 4 |
|  | *- Life not worth living* | 353 (98) | 3.01 | 3 | 1.06 | 0 | 4 |
|  | *- Enjoyment* | 353 (98) | 1.94 | 2 | 0.89 | 0 | 4 |
|  | - Hopeful about future | 353 (98) | 1.66 | 2 | 0.99 | 0 | 4 |
|  | *- Loneliness* | 353 (98) | 1.63 | 2 | 1.17 | 0 | 4 |
|  | *- Confidence* | 353 (98) | 1.27 | 1 | 0.99 | 0 | 4 |
|  | *- Physical health* | 353 (98) | 3.10 | 3 | 0.92 | 0 | 4 |
|  | Summary score^1^ | 353 (98) | 18.60 | 18 | 6.40 | 3 | 37 |
|  | ReQoL-UI^1^ | 353 (98) | 0.778 | 1 | 0.141 | 0.115 | 0.995 |

**Acronyms.** EQ-5D-5L, EQ-5D five-level version; GAD-7, Generalized Anxiety Disorder-7 item; PHQ-9, Patient Health Questionnaire-9 item; ReQoL-10, Recovering Quality of Life – 10 item; ReQoL-UI, Recovering Quality of Life – Utility Index.

^1^ ReQoL-10 summary score does not include the physical health item. ReQoL-UI tariff based on 7 items: unable to cope (Q3), happiness (Q5), Life not worth living (Q6), enjoyment (Q7), loneliness (Q9), confidence (Q10), and physical health (Q11). Those items included in the ReQoL-UI are presented in *italics* in the above table.

**Table S6.2: Correlation coefficient matrix between EQ-5D-5L, ReQoL-10/-UI, and PHQ-9 items, summary, and preference-based scores at baseline**

|  | **Spearman’s rank correlation coefficient (p-value)** | | | | | | | | | |
| --- | --- | --- | --- | --- | --- | --- | --- | --- | --- | --- |
| **Measures** | **PHQ-9** | | | | | | | | | |
|  | Interest/ pleasure  in doing things | Down/  depressed/ hopeless | Sleep | Tired/  energy | Poor  appetite/  overeating | Feeling  bad/  failure | Concentration | Moving or  speaking slowly  OR fidgety/restless | Suicidal/  self-harm  thoughts | Summary score |
| **EQ-5D-5L** |  |  |  |  |  |  |  |  |  |  |
| - Mobility | 0.039 (0.471) | 0.102 (0.056) | 0.063 (0.242) | 0.087 (0.103) | 0.006 (0.914) | 0.058 (0.276) | -0.011 (0.831) | 0.066 (0.214) | 0.088 (0.099) | 0.083 (0.121) |
| - Self-care | 0.196 (<0.001) | 0.234 (<0.001) | 0.118 (0.026) | 0.121 (0.023) | 0.141 (0.008) | 0.131 (0.014) | 0.121 (0.023) | 0.128 (0.017) | 0.210 (<0.001) | 0.249 (<0.001) |
| - Usual activities | 0.305 (<0.001) | 0.235 (<0.001) | 0.247 (<0.001) | 0.200 (<0.001) | 0.197 (<0.001) | 0.165 (0.002) | 0.282 (<0.001) | 0.156 (0.003) | 0.140 (0.008) | 0.337 (<0.001) |
| - Pain/ discomfort | 0.070 (0.191) | 0.102 (0.056) | 0.092 (0.086) | 0.067 (0.212) | 0.091 (0.089) | 0.059 (0.268) | 0.076 (0.154) | 0.119 (0.025) | 0.106 (0.047) | 0.131 (0.014) |
| - Anxiety/ depression | 0.284 (<0.001) | 0.295 (<0.001) | 0.169 (0.001) | 0.185 (<0.001) | 0.199 (<0.001) | 0.239 (<0.001) | 0.229 (<0.001) | 0.209 (<0.001) | 0.140 (0.008) | 0.346 (<0.001) |
| VSE | -0.303 (<0.001) | -0.320 (<0.001) | -0.232 (<0.001) | -0.214 (<0.001) | -0.232 (<0.001) | -0.228 (<0.001) | -0.261 (<0.001) | -0.241 (<0.001) | -0.182 (0.001) | -0.391 (<0.001) |
| Cross-walk | -0.295 (<0.001) | -0.309 (<0.001) | -0.225 (<0.001) | -0.213 (<0.001) | -0.221 (<0.001) | -0.225 (<0.001) | -0.257 (<0.001) | -0.233 (<0.001) | -0.177 (0.001) | -0.382 (<0.001) |
| **ReQoL-10*/-UI***^1^ |  |  |  |  |  |  |  |  |  |  |
| - Starting daily tasks | -0.530 (<0.001) | -0.351 (<0.001) | -0.273 (<0.001) | -0.315 (<0.001) | -0.295 (<0.001) | -0.259 (<0.001) | -0.346 (<0.001) | -0.221 (<0.001) | -0.135 (0.011) | -0.493 (<0.001) |
| - Trust others | -0.089 (0.097) | -0.126 (0.018) | 0.028 (0.598) | -0.135 (0.011) | -0.204 (<0.001) | -0.140 (0.009) | -0.105 (0.050) | -0.082 (0.126) | -0.088 (0.098) | -0.160 (0.003) |
| *- Unable to cope* | *-0.328 (<0.001)* | *-0.314 (<0.001)* | *-0.208 (<0.001)* | *-0.262 (<0.001)* | *-0.216 (<0.001)* | *-0.284 (<0.001)* | *-0.291 (<0.001)* | *-0.224 (<0.001)* | *-0.223 (<0.001)* | *-0.425 (<0.001)* |
| - Doing things | -0.345 (<0.001) | -0.277 (<0.001) | -0.197 (<0.001) | -0.213 (<0.001) | -0.240 (<0.001) | -0.202 (<0.001) | -0.270 (<0.001) | -0.203 (<0.001) | -0.155 (0.003) | -0.378 (<0.001) |
| *- Happy* | *-0.361 (<0.001)* | *-0.364 (<0.001)* | *-0.183 (0.001)* | *-0.214 (<0.001)* | *-0.258 (<0.001)* | *-0.270 (<0.001)* | *-0.241 (<0.001)* | *-0.147 (0.006)* | *-0.167 (0.002)* | *-0.380 (<0.001)* |
| *- Life not worth living* | *-0.235 (<0.001)* | *-0.288 (<0.001)* | *-0.130 (0.014)* | *-0.106 (0.046)* | *-0.212 (<0.001)* | *-0.338 (<0.001)* | *-0.234 (<0.001)* | *-0.193 (<0.001)* | *-0.577 (<0.001)* | *-0.402 (<0.001)* |
| *- Enjoyment* | *-0.354 (<0.001)* | *-0.323 (<0.001)* | *-0.157 (0.003)* | *-0.251 (<0.001)* | *-0.223 (<0.001)* | *-0.262 (<0.001)* | *-0.227 (<0.001)* | *-0.163 (0.002)* | *-0.217 (<0.001)* | *-0.371 (<0.001)* |
| - Hopeful about future | -0.345 (<0.001) | -0.285 (<0.001) | -0.213 (<0.001) | -0.271 (<0.001) | -0.202 (<0.001) | -0.290 (<0.001) | -0.246 (<0.001) | -0.150 (0.005) | -0.235 (<0.001) | -0.391 (<0.001) |
| *- Loneliness* | *-0.179 (0.001)* | *-0.243 (<0.001)* | *-0.213 (<0.001)* | *-0.102 (0.056)* | *-0.215 (<0.001)* | *-0.247 (<0.001)* | *-0.167 (0.002)* | *-0.143 (0.007)* | *-0.192 (<0.001)* | *-0.302 (<0.001)* |
| *- Confidence* | *-0.287 (<0.001)* | *-0.373 (<0.001)* | *-0.186 (<0.001)* | *-0.170 (0.001)* | *-0.258 (<0.001)* | *-0.387 (<0.001)* | *-0.242 (<0.001)* | *-0.153 (0.004)* | *-0.211 (<0.001)* | *-0.411 (<0.001)* |
| *- Physical health* | *-0.137 (0.010)* | *-0.182 (0.001)* | *-0.158 (0.003)* | *-0.164 (0.002)* | *-0.077 (0.152)* | *-0.062 (0.243)* | *-0.100 (0.061)* | *-0.110 (0.040)* | *-0.047 (0.381)* | *-0.176 (0.001)* |
| Summary score^1^ | -0.464 (<0.001) | -0.459 (<0.001) | -0.265 (<0.001) | -0.297 (<0.001) | -0.352 (<0.001) | -0.431 (<0.001) | -0.356 (<0.001) | -0.253 (<0.001) | -0.359 (<0.001) | -0.576 (<0.001) |
| *ReQoL-UI^1^* | *-0.404 (<0.001)* | *-0.443 (<0.001)* | *-0.290 (<0.001)* | *-0.284 (<0.001)* | *-0.301 (<0.001)* | *-0.364 (<0.001)* | *-0.318 (<0.001)* | *-0.268 (<0.001)* | *-0.308 (<0.001)* | *-0.529 (<0.001)* |

**Acronyms.** EQ-5D-5L, EQ-5D five-level version; PHQ-9, Patient Health Questionnaire-9 item; ReQoL-10, Recovering Quality of Life – 10 item; ReQoL-UI, Recovering Quality of Life – Utility Index.

**Cohens cut offs:** weak, <0.3; moderate, 0.3<0.5; strong, ≥ 0.5.

^1^ ReQoL-10 summary score does not include the physical health item. ReQoL-UI tariff based on 7 items: unable to cope (Q3), happiness (Q5), life not worth living (Q6), enjoyment (Q7), loneliness (Q9), confidence (Q10), and physical health (Q11). Those items included in the ReQoL-UI are presented in *italics* in the above table.

**Table S6.3: Correlation coefficient matrix between EQ-5D-5L, ReQoL-10/-UI, and GAD-7 items, summary, and preference-based scores at baseline**

|  | **Spearman’s rank correlation coefficient (p-value)** | | | | | | | |
| --- | --- | --- | --- | --- | --- | --- | --- | --- |
| **Measures** | **GAD-7** | | | | | | | |
|  | Nervous/ anxious | Stop/ control worrying | Worrying about things | Trouble relaxing | Restless | Annoyed/  irritable | Afraid | Summary score |
| **EQ-5D-5L** |  |  |  |  |  |  |  |  |
| - Mobility | 0.003 (0.951) | 0.024 (0.652) | 0.025 (0.640) | 0.094 (0.077) | 0.089 (0.095) | 0.070 (0.189) | 0.072 (0.176) | 0.069 (0.199) |
| - Self-care | 0.061 (0.257) | 0.013 (0.813) | -0.018 (0.730) | 0.070 (0.192) | 0.055 (0.302) | 0.017 (0.748) | 0.058 (0.278) | 0.054 (0.314) |
| - Usual activities | 0.118 (0.027) | 0.059 (0.266) | 0.048 (0.366) | 0.100 (0.062) | 0.090 (0.091) | 0.096 (0.072) | 0.040 (0.451) | 0.109 (0.042) |
| - Pain/ discomfort | 0.060 (0.260) | 0.085 (0.112) | 0.141 (0.008) | 0.132 (0.013) | 0.134 (0.012) | 0.118 (0.027) | 0.168 (0.002) | 0.169 (0.001) |
| - Anxiety/ depression | 0.411 (<0.001) | 0.395 (<0.001) | 0.426 (<0.001) | 0.378 (<0.001) | 0.326 (<0.001) | 0.338 (<0.001) | 0.299 (<0.001) | 0.514 (<0.001) |
| VSE | -0.301 (<0.001) | -0.283 (<0.001) | -0.324 (<0.001) | -0.306 (<0.001) | -0.297 (<0.001) | -0.255 (<0.001) | -0.275 (<0.001) | -0.408 (<0.001) |
| Cross-walk | -0.304 (<0.001) | -0.283 (<0.001) | -0.335 (<0.001) | -0.313 (<0.001) | -0.296 (<0.001) | -0.250 (<0.001) | -0.275 (<0.001) | -0.411 (<0.001) |
| **ReQoL-10*/-UI*** |  |  |  |  |  |  |  |  |
| - Starting daily tasks | -0.166 (0.002) | -0.133 (0.012) | -0.212 (<0.001) | -0.213 (<0.001) | -0.175 (0.001) | -0.171 (0.001) | -0.033 (0.543) | -0.223 (<0.001) |
| - Trust others | -0.106 (0.046) | -0.122 (0.022) | -0.089 (0.094) | -0.120 (0.024) | -0.053 (0.321) | -0.135 (0.011) | -0.105 (0.048) | -0.153 (0.004) |
| *- Unable to cope* | *-0.197 (<0.001)* | *-0.194 (<0.001)* | *-0.183 (0.001)* | *-0.228 (<0.001)* | *-0.218 (<0.001)* | *-0.209 (<0.001)* | *-0.121 (0.023)* | *-0.263 (<0.001)* |
| - Doing things | -0.176 (0.001) | -0.121 (0.023) | -0.120 (0.025) | -0.259 (<0.001) | -0.165 (0.002) | -0.208 (<0.001) | -0.057 (0.282) | -0.221 (<0.001) |
| *- Happy* | *-0.091 (0.089)* | *-0.137 (0.010)* | *-0.150 (0.005)* | *-0.143 (0.007)* | *-0.132 (0.013)* | *-0.174 (0.001)* | *-0.045 (0.402)* | *-0.180 (0.001)* |
| *- Life not worth living* | *-0.156 (0.003)* | *-0.060 (0.259)* | *-0.083 (0.121)* | *-0.117 (0.028)* | *-0.119 (0.026)* | *-0.120 (0.025)* | *-0.112 (0.035)* | *-0.155 (0.004)* |
| *- Enjoyment* | *-0.152 (0.004)* | *-0.120 (0.024)* | *-0.138 (0.009)* | *-0.246 (<0.001)* | *-0.114 (0.033)* | *-0.158 (0.003)* | *-0.046 (0.392)* | *-0.197 (<0.001)* |
| - Hopeful about future | -0.094 (0.079) | -0.105 (0.048) | -0.142 (0.008) | -0.167 (0.002) | -0.156 (0.003) | -0.133 (0.012) | -0.057 (0.285) | -0.174 (0.001) |
| *- Loneliness* | *-0.127 (0.017)* | *-0.152 (0.004)* | *-0.213 (<0.001)* | *-0.202 (<0.001)* | *-0.143 (0.007)* | *-0.107 (0.046)* | *-0.094 (0.078)* | *-0.213 (<0.001)* |
| *- Confidence* | *-0.205 (<0.001)* | *-0.198 (<0.001)* | *-0.238 (<0.001)* | *-0.211 (<0.001)* | *-0.161 (0.002)* | *-0.246 (<0.001)* | *-0.135 (0.011)* | *-0.287 (<0.001)* |
| *- Physical health^1^* | *-0.089 (0.095)* | *-0.118 (0.027)* | *-0.161 (0.003)* | *-0.174 (0.001)* | *-0.130 (0.014)* | *-0.095 (0.077)* | *-0.148 (0.005)* | *-0.178 (0.001)* |
| Summary score^1^ | -0.242 (<0.001) | -0.223 (<0.001) | -0.264 (<0.001) | -0.302 (<0.001) | -0.217 (<0.001) | -0.252 (<0.001) | -0.130 (0.015) | -0.331 (<0.001) |
| *ReQoL-UI^1^* | *-0.216 (<0.001)* | *-0.230 (<0.001)* | *-0.266 (<0.001)* | *-0.309 (<0.001)* | *-0.242 (<0.001)* | *-0.238 (<0.001)* | *-0.186 (<0.001)* | *-0.339 (<0.001)* |

**Acronyms.** EQ-5D-5L, EQ-5D five-level version; GAD-7, Generalized Anxiety Disorder-7 item; ReQoL-10, Recovering Quality of Life – 10 item; ReQoL-UI, Recovering Quality of Life – Utility Index.

**Cohen’s ACS cut offs:** weak, <0.3; moderate, 0.3<0.5; strong, ≥ 0.5.

^1^ ReQoL-10 summary score does not include the physical health item. ReQoL-UI tariff based on 7 items: unable to cope (Q3), happiness (Q5), life not worth living (Q6), enjoyment (Q7), loneliness (Q9), confidence (Q10), and physical health (Q11). Those items included in the ReQoL-UI are presented in *italics* in the above table.

# **Appendix S7: Beyond the GAD-7 and PHQ-9 – additional analyses using the IAPT-PS and WSAS**

**Background.** The PHQ-9 and GAD-7 measures represent two commonly used, patient-reported measures of depression severity and anxiety severity, respectively. Within IAPT services, other measures are routinely collected to capture other condition-specific and functional aspects including the IAPT Phobia Scales (IAPT-PS) and Work and Social Adjustment (WSAS) measures.

**Objective.** To further assess the construct validity of the EQ-5D-5L (VSE and cross-walk) and ReQoL-UI/-10 scores against the IAPT-PS and WSAS.

**Additional Measures.** The following two condition-specific and functioning measures were included to complement the analysis conducted using the GAD-7 and PHQ-9, as they are commonly, routinely collected measures within IAPT services (see also Table S7.1):

1. **IAPT Phobia Scales (IAPT-PS) [4].** Designed to capture patients scoring below PHQ-9 and GAD-7 clinical cut-offs, but whose lives may be significantly impaired by social anxiety, agoraphobia, and specific phobias.
2. **Work and Social Adjustment (WSAS) [5].** 5-item, self-reported measure of impaired functioning across: work; social life; home life; private life; close relationships.

**Methods.** The same methods are used as for assessing construct validity against the PHQ-9 and GAD-7 as described in the main manuscript.

**Results.** IAPT-PS and WSAS scores at baseline across trial-arms are presented in Table S7.2, and across time-point by trial-arm in Table S7.3. When assessing construct validity, all ACS and AES are presented in Table S7.4 and Table S7.5, respectively.

When comparing against the IAPT-PS, all ACS were weak, apart from ReQoL-UI/-10 with social anxiety (moderate). ReQoL-UI AES were medium (social anxiety and panic disorder) or small (specific phobia); these were weaker for the ReQoL-10 (panic disorder, small; specific phobia, trivial and not SS) apart from the social anxiety scale (medium). EQ-5D-5L (VSE and cross-walk) scores and anxiety/depression item had similar, small AES, apart for cross-walk and anxiety/depression item with panic disorder (medium); AES were larger for the cross-walk than VSE tariff and anxiety/depression item.

When comparing against the WSAS, all ACS were moderate, except with EQ-5D-5L item (weak). AES were: ReQoL-UI/-10, medium; EQ-5D-5L (VSE and cross-walk), similar, medium and small (dependent on WSAS severity); anxiety/depression item, small. EQ-5D-5L (VSE and cross-walk) and anxiety/depression item AES were stronger for moderately severe or worse psychopathology relative to significant functional impairment (but less severe clinical symptomatology); however, ReQoL-UI/-10 AES were stronger for significant functional impairment relative to subclinical.

**Conclusion.** The IAPT-PS measures are designed to capture patients scoring below PHQ-9 and GAD-7 clinical cut-offs, for which there were few patients in this group for this case study. This is in part represented in the IAPT-PSs score distributions and high ceiling effects, which limits the use of this measure for the intended construct validity analysis; therefore, the results are presented for the interested reader but should be interpreted with caution.

When compared with the WSAS, the ReQoL-UI/-10 had the better ACS and AES than the EQ-5D-5L, suggesting the ReQoL-UI/-10 has better construct validity with work and social functioning than the EQ-5D-5L VSE and cross-walk scores.

**Table S7.1: Description of outcomes measures and associated scores**

| **Long name** | **Short Name** | **Construct** | **Scoring type** | **No. items** | **Item score** | **Floor/**  **worst** | **Ceiling/**  **best** | **Cut-offs** | **Ref** |
| --- | --- | --- | --- | --- | --- | --- | --- | --- | --- |
| **Condition-specific** |  |  |  |  |  |  |  |  |  |
| IAPT Phobia Scales | IAPT-PS | Phobias | Q1 social anxiety;  Q2 panic disorder;  Q3 specific phobia | 1  (per Q) | 9-point scale per Q:  0 (wont avoid it) to  9 (always avoid it) | 8  (per Q) | 0  (per Q) | <4, No caseness;  ≥4, Caseness  (per Q) | [4] |
| **Functional Impairment** | |  |  |  |  |  |  |  |  |
| Work and Social Adjustment Scale | WSAS^1^ | Work and social functioning | Summary | 5 | 9-point scale:  0 (not at all) to  8 (very severely) | 40 | 0 | <10, Subclinical;  10-20, Func. impaired;  ≥21, Mod./Sev. Psychopathology | [5] |

^1^ **WSAS**: shortened terminology are used for the cut-offs for descriptive purposes only; the full cut-off descriptions (score range) are: Subclinical population (<10); significant functional impairment but less severe clinical symptomatology (10-20); moderately severe or worse psychopathology (≥21).

**Table S7.2: Outcome measure scores, floor and ceiling effects at baseline across trial-arms**

| **Short Name** | **N (%)** | **Mean** | **Median** | **SD** | **P. floor/**  **worst score** | **P. ceiling/**  **best score** | **O.**  **worst score** | **O.**  **best score** | **N floor/**  **worst score (%)** | **N ceiling/**  **best score (%)** |
| --- | --- | --- | --- | --- | --- | --- | --- | --- | --- | --- |
| **Condition specific** |  |  |  |  |  |  |  |  |  |  |
| IAPT-PS social anxiety | 361 (100) | 3.216 | 3 | 2.224 | 8 | 0 | 8 | 0 | 7 (1.9) | 55 (15.2) |
| IAPT-PS panic disorder | 361 (100) | 2.468 | 2 | 2.414 | 8 | 0 | 8 | 0 | 12 (3.3) | 114 (31.6) |
| IAPT-PS specific phobia | 361 (100) | 2.141 | 1 | 2.416 | 8 | 0 | 8 | 0 | 14 (3.9) | 146 (40.4) |
| **Functional impairment** |  |  |  |  |  |  |  |  |  |  |
| WSAS summary score | 361 (100) | 17.992 | 18 | 7.415 | 40 | 0 | 39 | 0 | 0 (0) | 2 (0.6) |

**Acronyms** IAPT-PS, Improving Access to Psychological Therapies - Phobia Scales^5^; N, number of responder; O., observed; P., possible; SD, standard deviation; WSAS, Work and Social Adjustment Scale.

**Footnote.** The table shows the possible (P.) floor/worst and ceiling/best scores as well as the observed (O.) worst and best scores achieved by the respondents; these are shown rather than possible and observed minimum and maximum scores due to the fact for the tariff scores a higher score is a better state, whereas for the summary scores the opposite is true (i.e. a higher score is a worst state)

**Table S7.3: Observed PROM scores, number of responders, and standardised response means by trial-arm and time points**

| **Measure** | t_i_ | **Intervention (I), N = 241** | | | | | | | | **Control (C), N = 120** | | | | |
| --- | --- | --- | --- | --- | --- | --- | --- | --- | --- | --- | --- | --- | --- | --- |
|  |  | **Time-point (t_i_)** | | **Dif. time-points, t_i_ – t_0_** | | | **Dif. time-points, t_i_ – t_i-1_** | | | **Time-point (t_i_)** | | **Dif. time-points, t_i_ – t_0_** | | |
|  |  | **N (%)** | **Mean (SD)** | **N (%)** | **Mean (SD)** | **SRM** | **N (%)** | **Mean (SD)** | **SRM** | **N (%)** | **Mean (SD)** | **N (%)** | **Mean (SD)** | **SRM** |
| IAPT-PS | t_0_ | 241 (100) | 3.14 (2.18) | - | - | - | - | - | - | 120 (100) | 3.37 (2.32) | - | - | - |
| social | t_1_ | 198 (82) | 2.33 (1.95) | 198 (82) | -0.79 (1.86) | -0.425 | 198 (82) | -0.79 (1.86) | -0.425 | 91 (76) | 3.53 (2.39) | 91 (76) | 0.07 (2.24) | 0.029 |
| anxiety | t_2_ | 186 (77) | 2.12 (1.99) | 186 (77) | -1.02 (2.02) | -0.503 | 177 (73) | -0.22 (1.21) | -0.182 | - | - | - | - | - |
| item | t_3_ | 182 (76) | 1.98 (1.98) | 182 (76) | -1.08 (2.13) | -0.509 | 169 (70) | -0.11 (1.55) | -0.073 | - | - | - | - | - |
| score | t_4_ | 176 (73) | 1.84 (1.81) | 176 (73) | -1.31 (2.11) | -0.620 | 166 (69) | -0.22 (1.49) | -0.150 | - | - | - | - | - |
|  | t_5_ | 173 (72) | 1.85 (1.92) | 173 (72) | -1.25 (2.43) | -0.514 | 160 (66) | 0.04 (1.56) | 0.028 | - | - | - | - | - |
| IAPT-PS | t_0_ | 241 (100) | 2.31 (2.36) | - | - | - | - | - | - | 120 (100) | 2.78 (2.50) | - | - | - |
| panic | t_1_ | 198 (82) | 1.73 (1.94) | 198 (82) | -0.49 (1.80) | -0.270 | 198 (82) | -0.49 (1.80) | -0.270 | 91 (76) | 2.59 (2.51) | 91 (76) | -0.25 (2.21) | -0.114 |
| disorder | t_2_ | 186 (77) | 1.56 (1.83) | 186 (77) | -0.66 (1.76) | -0.377 | 177 (73) | -0.16 (1.22) | -0.134 | - | - | - | - | - |
| item | t_3_ | 182 (76) | 1.48 (1.98) | 182 (76) | -0.76 (1.96) | -0.390 | 169 (70) | -0.11 (1.33) | -0.084 | - | - | - | - | - |
| score | t_4_ | 176 (73) | 1.47 (1.94) | 176 (73) | -0.80 (2.03) | -0.396 | 166 (69) | -0.11 (1.34) | -0.086 | - | - | - | - | - |
|  | t_5_ | 173 (72) | 1.41 (1.87) | 173 (72) | -0.85 (2.06) | -0.412 | 160 (66) | -0.04 (1.53) | -0.024 | - | - | - | - | - |
| IAPT-PS | t_0_ | 241 (100) | 2.10 (2.38) | - | - | - | - | - | - | 120 (100) | 2.23 (2.50) | - | - | - |
| specific | t_1_ | 198 (82) | 1.64 (1.92) | 198 (82) | -0.48 (1.83) | -0.260 | 198 (82) | -0.48 (1.83) | -0.260 | 91 (76) | 2.44 (2.29) | 91 (76) | 0.14 (2.10) | 0.068 |
| phobia | t_2_ | 186 (77) | 1.47 (1.87) | 186 (77) | -0.60 (1.90) | -0.317 | 177 (73) | -0.12 (1.28) | -0.097 | - | - | - | - | - |
| item | t_3_ | 182 (76) | 1.39 (1.92) | 182 (76) | -0.76 (2.18) | -0.347 | 169 (70) | -0.11 (1.47) | -0.077 | - | - | - | - | - |
| score | t_4_ | 176 (73) | 1.38 (1.82) | 176 (73) | -0.74 (2.05) | -0.360 | 166 (69) | -0.07 (1.38) | -0.052 | - | - | - | - | - |
|  | t_5_ | 173 (72) | 1.42 (1.94) | 173 (72) | -0.75 (2.10) | -0.357 | 160 (66) | -0.04 (1.34) | -0.028 | - | - | - | - | - |
| WSAS | t_0_ | 241 (100) | 17.32 (7.12) | - | - | - | - | - | - | 120 (100) | 19.34 (7.84) | - | - | - |
| summary | t_1_ | 198 (82) | 12.70 (8.31) | 198 (82) | -4.74 (8.07) | -0.587 | 198 (82) | -4.74 (8.07) | -0.587 | 91 (76) | 16.90 (8.57) | 91 (76) | -1.55 (8.09) | -0.192 |
| score | t_2_ | 186 (77) | 11.76 (8.30) | 186 (77) | -6.02 (8.18) | -0.735 | 177 (73) | -1.03 (5.12) | -0.201 | - | - | - | - | - |
|  | t_3_ | 182 (76) | 10.30 (8.72) | 182 (76) | -7.01 (9.22) | -0.760 | 169 (70) | -1.28 (7.06) | -0.181 | - | - | - | - | - |
|  | t_4_ | 176 (73) | 10.01 (8.62) | 176 (73) | -7.48 (9.66) | -0.775 | 166 (69) | -0.49 (6.59) | -0.074 | - | - | - | - | - |
|  | t_5_ | 172 (71) | 9.90 (8.44) | 172 (71) | -7.53 (9.52) | -0.791 | 159 (66) | -0.24 (6.26) | -0.038 | - | - | - | - | - |

**Acronyms.** IAPT-PS, Improving Access to Psychological Therapies - Phobia Scales^5^; N, Number of people; SD, Standard Deviation; SRM, Standardised Response Mean; WSAS, Work and Social Adjustment Scale.

**Labelling.** t = time point, whereby: t_0_ = baseline; t_1_ = 8 weeks; t_2_ = 3 months; t_3_ = 6 months; t_4_ = 9 months; t_5_ = 12 months.

**Footnote.** N(%) states the number of people who completed the measure at the specific time-point, or at two given time-points e.g. relative to t_0_ (baseline) or t_i_ (whereby i is any time-point denoted as 1 to 5).

**Cohens SRM cut off:** <0.2, trivial; 0.2< 0.5, small; 0.5 < 0.8, medium; ≥0.8, large; an SRM of >1 means the change in score between time-points is larger than one standard deviation. ESs are relative to less severe group.

**Table S7.4: Correlation coefficient matrix between outcome measures at baseline**

| **Measures** | **Spearman’s rank correlation coefficient (p-value)** | | | | |
| --- | --- | --- | --- | --- | --- |
|  | EQ-5D-5L  VSE | EQ-5D-5L  cross-walk | ReQoL-UI | ReQoL-10 | EQ-5D-5L  depression/anxiety |
| **Condition-specific** |  |  |  |  |  |
| IAPT-PS social anxiety | -0.242 (<0.001) | -0.240 (<0.001) | -0.333 (<0.001) | -0.353 (<0.001) | 0.232 (<0.001) |
| IAPT-PS panic disorder | -0.269 (<0.001) | -0.270 (<0.001) | -0.227 (<0.001) | -0.219 (<0.001) | 0.287 (<0.001) |
| IAPT-PS specific phobia | -0.193 (<0.001) | -0.187 (<0.001) | -0.183 (0.001) | -0.108 (0.042) | 0.167 (0.002) |
| **Functional impairment** |  |  |  |  |  |
| WSAS summary score | -0.343 (<0.001) | -0.342 (<0.001) | -0.456 (<0.001) | -0.451 (<0.001) | 0.231 (<0.001) |

**Acronyms.** EQ-5D-5L, EQ-5D five-level version; IAPT-PS, Improving Access to Psychological Therapies - Phobia Scales; ReQoL-10, Recovering Quality of Life – 10 item; ReQoL-UI, Recovering Quality of Life – Utility Index.

**Cohen’s ACS cut-offs:** weak, <0.3; moderate, 0.3<0.5; strong, ≥ 0.5; p-value < 0.05.

**Table S7.5: Testing known-group validity between generic health status measures and condition-specific or functionality measures at baseline**

| **Measures** |  |  | **EQ-5D-5L**  **VSE** | | **EQ-5D-5L**  **cross-walk** | | **ReQoL-UI** | | **ReQoL-10** | | **EQ-5D-5L**  **depression/anxiety** | |
| --- | --- | --- | --- | --- | --- | --- | --- | --- | --- | --- | --- | --- |
|  | Groups,  score range | N (%) | Mean (SD) | ES  (p-value) | Mean (SD) | ES  (p-value) | Mean (SD) | ES  (p-value) | Mean (SD) | ES  (p-value) | Mean (SD) | ES  (p-value) |
| **Diagnosis specific** | |  |  |  |  |  |  |  |  |  |  |  |
| IAPT-PS | No Case., <4 | 211 (58.4) | 0.761 (0.144) |  | 0.693 (0.178) |  | 0.809 (0.111) |  | 20.162 (6.484) |  | 3.097 (0.797) |  |
| social | Caseness, ≥4 | 150 (41.6) | 0.687 (0.177) | 0.467 | 0.596 (0.219) | 0.499 | 0.735 (0.165) | 0.544 | 16.456 (5.638) | 0.603 | 3.483 (0.819) | -0.479 |
| anxiety |  |  |  | (<0.001) |  | (<0.001) |  | (<0.001) |  | (<0.001) |  | (<0.001) |
| IAPT-PS | No Case., <4 | 242 (67.0) | 0.755 (0.156) |  | 0.688 (0.183) |  | 0.802 (0.110) |  | 19.346 (6.261) |  | 3.126 (0.762) |  |
| panic | Caseness, ≥4 | 119 (33.0) | 0.679 (0.165) | 0.481 | 0.579 (0.218) | 0.560 | 0.729 (0.181) | 0.532 | 17.069 (6.437) | 0.360 | 3.534 (0.889) | -0.508 |
| disorder |  |  |  | (<0.001) |  | (<0.001) |  | (<0.001) |  | (0.001) |  | (<0.001) |
| IAPT-PS | No Case., <4 | 267 (74.0) | 0.744 (0.160) |  | 0.672 (0.193) |  | 0.792 (0.125) |  | 18.826 (6.189) |  | 3.187 (0.811) |  |
| specific | Caseness, ≥4 | 94 (26.0) | 0.691 (0.164) | 0.328 | 0.596 (0.215) | 0.383 | 0.739 (0.173) | 0.379 | 17.968 (6.948) | 0.134 | 3.462 (0.841) | -0.336 |
| phobia |  |  |  | (0.003) |  | (0.002) |  | (0.003) |  | (0.133) |  | (0.019) |
| **Functional impairment** | |  |  |  |  |  |  |  |  |  |  |  |
| WSAS | Subclinical, <10 | 48 (13.3) | 0.802 (0.113) |  | 0.743 (0.128) |  | 0.868 (0.070) |  | 24.178 (6.271) |  | 2.978 (0.715) |  |
| summary | Func. Imp., 10-20 | 188 (52.1) | 0.757 (0.146) | 0.325 | 0.679 (0.196) | 0.346 | 0.797 (0.109) | 0.698 | 19.054 (5.902) | 0.858 | 3.189 (0.788) | -0.272 |
| score | Mod./Sev. Psy., ≥21 | 125 (34.6) | 0.664 (0.180) | 0.579 | 0.578 (0.210) | 0.501 | 0.715 (0.174) | 0.588 | 15.870 (5.673) | 0.548 | 3.468 (0.878) | -0.337 |
|  |  |  |  | (<0.001) |  | (<0.001) |  | (<0.001) |  | (<0.001) |  | (0.001) |

**Acronyms.** Clin., clinical; ES, effect size (Cohens d); Func. Imp, significant functional impairment but less severe clinical symptomatology; Gen. Pop., General Population; Mod., Moderate; Mod. Sev., Moderately Severe; Mod. Sev. Psy., moderately severe or worse psychopathology; SD, Standard Deviation; Sev., Severe.

**Cohen’s AES cut-off:** trivial, <0.2; small, 0.2< 0.5; medium, 0.5 < 0.8; large; ≥0.8; an ES of >1 means the difference between the two means is larger than one standard deviation. ESs are relative to less severe.

**P-values:** calculated from the non-parametric Kruskal Wallis test to suggest if there is a statistically significant difference between two or more known-groups based on the scores used as a complement to assessing ES.

# **References**

1. Richards, D., Enrique, A., Eilert, N., Franklin, M., Palacios, J., Duffy, D., Earley, C., Chapman, J., Jell, G., & Sollesse, S. (2020). A pragmatic randomized waitlist-controlled effectiveness and cost-effectiveness trial of digital interventions for depression and anxiety. NPJ digital medicine, 3(1), 1-10.

2. Cleveland, W. S. (1979). Robust locally weighted regression and smoothing scatterplots. Journal of the American statistical association, 74(368), 829-836.

3. Cohen, J. (1992). A power primer. Psychological bulletin, 112(1), 155.

4. NHS. (2011). The Improving Access to Psychological Therapies Data Handbook v2.0.1. In N. H. S. (NHS) (Ed.). London.

5. Mundt, J. C., Marks, I. M., Shear, M. K., & Greist, J. M. (2002). The Work and Social Adjustment Scale: a simple measure of impairment in functioning. The British Journal of Psychiatry, 180(5), 461-464.
